# *Oncorhynchus mykiss* silage improves meat fatty acids profile, blood parameters, intestinal histomorphometry, productive performance, and modulates the cecal microbiota of *Cavia porcellus*

**DOI:** 10.3389/fnut.2025.1725233

**Published:** 2026-01-06

**Authors:** Miguel Arista, Segundo José Zamora-Huamán, José Américo Saucedo-Uriarte, Paul Fernandez-Castro, Niger Maldonado, Loidy Valle, Jakson Ch. Del Solar, Diana L. Arista Vargas, Rainer M. Lopez Lapa, Carlos Torres, Yoany Leiva, Héctor V. Vásquez, Jorge L. Maicelo, William Bardales

**Affiliations:** 1 Grupo de Investigación en Nutrición y Alimentación de Animales de Interés Zootécnico, Instituto de Investigación en Ganadería y Biotecnología, Facultad de Ingeniería Zootecnista, Biotecnología, Agronegocios y Ciencia de Datos, Universidad Nacional Toribio Rodríguez de Mendoza de Amazonas, Chachapoyas, Peru; 2 Instituto de Investigación en Ganadería y Biotecnología, Facultad de Ingeniería Zootecnista, Biotecnología, Agronegocios y Ciencia de Datos, Universidad Nacional Toribio Rodríguez de Mendoza de Amazonas, Chachapoyas, Peru; 3 Laboratorio de Fisiología Molecular, Instituto de Investigación en Ganadería y Biotecnología, Facultad de Ingeniería Zootecnista, Biotecnología, Agronegocios y Ciencia de Datos, Universidad Nacional Toribio Rodríguez de Mendoza de Amazonas, Chachapoyas, Peru; 4 Escuela Profesional de Medicina Humana, Facultad de Medicina, Universidad Nacional Toribio Rodríguez de Mendoza de Amazonas, Chachapoyas, Peru; 5 Grupo de Investigación en Bromatología de los Alimentos, Instituto de Investigación en Ganadería y Biotecnología, Facultad de Ingeniería Zootecnista, Biotecnología, Agronegocios y Ciencia de Datos, Universidad Nacional Toribio Rodríguez de Mendoza de Amazonas, Chachapoyas, Peru; 6 Laboratorio de Agrostología, Instituto de Investigación en Ganadería y Biotecnología, Facultad de Ingeniería Zootecnista, Biotecnología, Agronegocios y Ciencia de Datos, Universidad Nacional Toribio Rodríguez de Mendoza de Amazonas, Chachapoyas, Peru; 7 Laboratorio de Enfermedades Infecciosas y Parasitarias de Animales Domésticos, Instituto de Investigación en Ganadería y Biotecnología, Facultad de Ingeniería Zootecnista, Biotecnología, Agronegocios y Ciencia de Datos, Universidad Nacional Toribio Rodríguez de Mendoza de Amazonas, Chachapoyas, Peru

**Keywords:** domestic guinea pig, fish hydrolysate, circular economy, meat quality, genomic sequencing, productive performance, health

## Abstract

The use of rainbow trout silage represents an alternative for the feeding of domestic species such as guinea pigs with a circular economy approach. The objective of this study was to evaluate the effect of *Oncorhynchus mykiss* silage in the diet of *Cavia porcellus* on the fatty acid profile of the meat, blood parameters, intestinal histomorphometry, productive performance, and cecal microbiota. Thirty-three weaned male guinea pigs of the Peru breed were used, distributed in three treatments: T0 (0%), T1 (4%), and T2 (8% silage inclusion in the diet), with 11 replicates each. The inclusion of silage in the diets significantly modified the lipid profile of the meat, increasing monounsaturated fatty acids and docosahexaenoic acid (DHA, C22:6) (*p* < 0.001) compared to T0. No changes were observed in most hematological and biochemical parameters, with the exception of mean platelet volume (MPV) (*p* = 0.020) and total cholesterol (TC) (*p* < 0.001). Higher silage inclusion favored the development of crypt depth (*p* = 0.047). The alpha diversity of cecal microbiota did not vary (*p* > 0.05), but beta diversity revealed compositional changes associated with silage consumption (Bray–Curtis, *p* = 0.006; Jaccard, *p* = 0.017). Productive performance, such as weight gain increased in T2 (*p* = 0.050) and feed conversion factor, decreased with silage inclusion (*p* < 0.001), without affecting intake. In conclusion, trout silage represents a sustainable alternative ingredient in the use of fish by-products that improves the nutritional quality of guinea pig meat and optimizes feed efficiency, without compromising animal health or performance.

## 1 Introduction

*Cavia porcellus* is a South American rodent species of the family *Caviidae* and genus *Cavia* (1). Its breeding plays a very important role in rural families in Peru, Ecuador, Colombia, and Bolivia, where it is deeply rooted as a cultural practice, contributes to food security, and serves as a source of economic income (2, 3). Peru stands out as the primary producer (17.4 million animals) and per capita consumer of guinea pigs (0.66 kg/person/year), increasing over the years (4). The consumption of guinea pig meat is due to its high protein content and biological value (5).

Traditionally, guinea pig have been fed kitchen by-products and pasture as supplements (6). The growing demand for guinea pig meat requires the optimization of feeding and management programs, for which it is essential to determine nutritional requirements and evaluate the nutritional value of available feed inputs (7). The guinea pig has the capacity to adapt to diverse climatic conditions and ecosystems, due to its physiological capacities as a herbivorous species, its short reproductive cycle, and its versatile diet (2). The transition to more intensive breeding systems, the use of improved genetic lines, and the use of balanced feeds make the guinea pig activity more essential to meet their nutritional needs (8). Consequently, the need to produce naturally dense feeds with high nutritional content has led to the use of agricultural and industrial by-products, which are essential for maintaining productivity without increasing production costs (9), while also contributing to environmental sustainability (10).

Among the by-products of the fishing industry, fish silage has emerged as a promising ingredient. It contains bioactive compounds, fatty acids such as omega (*ω*)-3, hydrolyzed proteins, small peptides, and free amino acids that are rapidly digested and absorbed (11–13).

There is evidence on the use of silage sources as a dietary supplement in various species, observing positive impacts on growth, intestinal health, and lipid composition of meat (14–16). For example, in tilapia (*Oreochromis mossambicus*), the inclusion of rainbow trout (*Oncorhynchus mykiss*) silage oil effectively replaced fish oil by not affecting the composition of *ω*-3 polyunsaturated fatty acid (*ω*-3 PUFA) levels in fillets (17). Similarly, in South African abalone (*Haliotis midae*), the inclusion of *O. mykiss* silage oil in the feeding of has been shown to have no significant effects on immune function, proximal composition, and fatty acid profile of abalone muscle (18).

Furthermore, Güllü et al. (19) found that it is possible to replace up to 20% fish meal with fish silage in *O. mykiss* feed without negatively affecting growth, fatty acid composition, and serum biochemical parameters. Fish silage can promote a more optimal intestinal morphology by stimulating intestinal mucosal folds that are deeper and more regular, as well as maintaining the integrity of microvilli. This contributes to a better health status in fish, which is reflected in specific hematological parameters in tilapia (20).

Despite its demonstrated benefits in feeding aquaculture species, the use of fish silage in herbivorous animals such as guinea pigs has not yet been explored. This study provides new evidence on how trout silage can benefit the feeding of herbivorous monogastric animals, such as guinea pigs, with enzymatic digestion in the stomach and bacterial digestion in the cecum (2). Although *C. porcellus* has a herbivorous digestive physiology and relies on cecal fermentation to obtain energy from structural carbohydrates, fish possess a more active gastric phase and higher levels of pancreatic proteases and lipases (21). Therefore, the incorporation of marine-derived lipids or peptides from silage could have species-specific physiological consequences.

In recent years, the characterization of the intestinal microbiota in animals of zootechnical interest has advanced considerably due to modern molecular tools (22). The use of high-throughput sequencing platforms has facilitated a more accurate and in-depth view of bacterial diversity (23, 24). Sequencing of hypervariable regions of the 16S rRNA gene provides insight into how different factors, such as intestinal inflammation, parental offspring, and diet, can modify the microbial composition, potentially influencing intestinal health and productive efficiency in animals of zootechnical interest (25–27). Given the growing demand for functional foods of animal origin and the interest in increasingly sustainable production systems, it is pertinent to explore alternative ingredients that simultaneously improve the nutritional quality of the meat, the physiological parameters of the animal, and the productive efficiency. Despite the evidence accumulated in other species, the knowledge of the effects of fish silage, particularly of *O. mykiss* on *C. porcellus,* is scarce; therefore, the objective of this research was to evaluate the effect of *O. mykiss* silage on meat fatty acids, blood parameters, productive performance, intestinal histomorphometry, and cecal microbiota of guinea pigs of the Peruvian breed.

## 2 Materials and methods

### 2.1 Ethical statement

Animal welfare was ensured at all times during the research. The euthanasia process followed the guidelines of ARRIVE 2.0 and the AVMA Guide: 2020 Edition (28, 29). The study was approved by the Institutional Research Ethics Committee of the University Nacional Toribio Rodriguez de Mendoza of Amazonas, through code CIEI-N°0061.

### 2.2 Place of study

The research was carried out in the guinea pig module of the Chachapoyas Experimental Station of the Livestock and Biotechnology Research Institute of the Universidad Nacional Toribio Rodríguez de Mendoza de Amazonas (UNTRM), located in the southern hemisphere of the province of Chachapoyas, Amazonas region. The city of Chachapoyas is located in the northern Andes of Peru, at an altitude of 2,338 m.a.s.l., with an average annual rainfall of 800 mm and an average temperature of 15.6 °C. There is a very low annual thermal variation, with 1 °C in average monthly temperatures. The lowest monthly minimum temperature values (10.5 °C) are reported between June and August, while the highest monthly maximum temperature values (25.8 °C) occur from August to December (30).

### 2.3 Animals and management

Thirty-three weaned male guinea pigs of the Peru breed were selected, 15 ± 2 days old with an average weight of 248.35 ± 3.65 g, distributed in three treatments (T0: 0% inclusion of trout silage in the diet, T1: 4% inclusion of trout silage in the diet, and T2: 8% inclusion of trout silage in the diet) with 11 replicates each. The research period lasted 11 weeks. A maximum of 8% trout silage was used, as this provided the energy balance in the diet. Each animal was identified with numbers engraved on aluminum earrings and placed on the animal’s right ear pinna; they were individually placed in a pool with brick walls lined with cement, chip floor, measuring 0.8 m × 0.9 m × 0.45 m in width, length, and height, respectively. The area provided was more than enough for each animal for both the rearing and fattening stages (31). Feed supply (alfalfa + balanced feed) was provided once a day at 8 a.m. The balanced feed was supplied in clay feeders placed in the center of each pool, while the grass was thrown directly into each pen at 20% of each animal’s live weight (fresh basis). The supply of grass was maintained at 20% of each animal’s live weight to encourage the consumption of balanced feed; thus, adjustments were made weekly (32). All animals always had water freely available through an automatic nipple type waterer. Silage preparation was performed in an artisanal manner following the guidelines of Toppe et al. (33). The viscera of rainbow trout sold at the municipal market in Chachapoyas were collected, chopped into 3 cm pieces, washed with drinking water to remove food and fecal matter, and stored for 30 days in 20-L airtight containers with 3% of 85% formic acid added (Spectrum Chemical Mfg. Corp., USA). Chemical analysis was performed by standard procedures of the Association of Official Analytical Chemists (34). The composition and nutritional values of the experimental diets, pasture, and trout silage are presented in Table 1. The fatty acid profile of the trout silage was analyzed according to the official method 996.06 of the Association of Official Agricultural Chemists (35).

**Table 1:** Composition and nutritional values of the experimental diets, forage, and trout silage.

Ingredients (Kg)	T0	T1	T2	Alfalfa	Trout silage
Wheat bran	63.15	57.00	52.70	–	–
Corn	16.00	21.10	21.80	–	–
Molasses	2.00	2.00	2.00	–	–
Soybean cake	15.00	13.00	12.70	–	–
Soybean oil	1.00	0.00	0.00	–	–
Trout silage	0.00	4.00	8.00	–	–
DL-Methionine (99%)	0.12	0.15	0.18	–	–
L-Lysine (98.5%)	0.00	0.00	0.04	–	–
Calcium carbonate	2.30	2.30	2.30	–	–
Premix vitamins^a^	0.10	0.10	0.10	–	–
Choline chloride (60%)	0.10	0.10	0.10	–	–
Common salt	0.20	0.20	0.20	–	–
Vitamin C	0.03	0.03	0.03	–	–
Total	100.0	100.0	100.0	–	–
*Nutrient*	*Analyzed chemical composition* ^b^				
Dry matter %	88.45	80.77	74.83	20.5	93.16
Ashes %	8.29	9.26	6.37	8.68	4.22
Crude fat %	3.29	3.40	4.32	3.02	52.58
Crude protein %	12.76	13.66	14.3	19.17	33.38
Crude fiber %	13.05	7.05	6.75	24.84	n.d.
Nitrogen free extract %	53.83	50.48	45.22	40.45	n.d.
*Trout silage fatty acid profile (%)* ^c^	*Composition*				
Caprylic acid (C8:0)	0.2				
Myristic acid (C14:0)	0.99				
Palmitic acid (C16:0)	10.13				
Palmitoleic acid (C16:1)	1.5				
Heptadecanoic acid (C17:0)	0.12				
Stearic acid (C18:0)	3.32				
Oleic acid (C18:1 n9c)	12.63				
Linoleic acid (C18:2 n6c)	8.3				
Arachidic acid (C20:0)	0.13				
Eicosenoic acid (C20:1 Cis 11)	0.45				
Linolenic acid (C18:3 n3)	0.51				
Eicosadienoic acid (C20:2)	0.53				
Behenic acid (C22:0)	0.1				
Eicosatrienoic acid (C20:3)	0.19				
Nervonic acid (C24:1)	0.16				
Docosahexaenoic acid (C22:6)	0.38				
Total fat	41.74				
Saturated fats	15.08				
Monounsaturated fats	15.11				
Polyunsaturated fats	9.91				

a Per kg of feed: 104 International Units (IU) Vitamin A; 2,500 IU Vitamin D3; 15 mg Vitamin E; 3 mg Vitamin K3; 1 mg Vitamin B1; 4 mg Vitamin B2; 3 mg Vitamin B6; 15 mg Vitamin B12; 8 mg Pantothenic Ac. B5; 0.5 mg Folic Acid (Vit. B9); 30 mg Nicotinic Acid (Vit. B3); 25 mcg biotin (Vit. B7); 7 mg choline; 5 mg copper; 25 mg iron; 60 mg manganese; 0.2 mg selenium; 0.5 mg iodine; 60 mg zinc; 1.3 g methionine.

b Laboratory of Animal Nutrition and Food Bromatology—National University Toribio Rodriguez of Mendoza of Amazonas.

c La Molina Total Quality Laboratories, National Agrarian University La Molina.

n.d., not determined.

### 2.4 Productive performance

Daily feed intake (g) was calculated by subtracting the feed fed on the previous day minus the residual feed, and the feed conversion factor (kg) was calculated as the ratio of total feed consumed on a fresh basis to total weight gained (36). Cumulative weight gain (g) was determined by the difference between the initial and the final live weights.

### 2.5 Hematological and biochemical analysis

The hematological analysis was performed at week 9 after starting the experiment. After a minimum fasting period of 10 h, 1.5 mL of blood was collected from the cranial vena cava, without prior anesthesia, into vacutainer tubes containing EDTA as anticoagulant (37). The following parameters were analyzed: white blood cell count, lymphocyte number, monocyte number, granulocyte number, lymphocyte percentage, red blood cell count, hemoglobin concentration, hematocrit, mean corpuscular volume, mean corpuscular hemoglobin, mean corpuscular hemoglobin concentration, platelet count, mean platelet volume, and procalcitonin at the Domestic Animal Infectious and Parasitic Diseases Laboratory. Samples were processed on a VH30 automated veterinary hematology analyzer (Shenzhen, China) following the methodology proposed by El-Moghazy et al. (38). For biochemical profiling, blood was collected in week 11 into tubes with clot activator (39). Serum was obtained by centrifugation at 825 × *g* for 10 min at room temperature (Rotofix 32A, Hettich, Germany). Within 5 days, the following were assessed using commercial kits (Quimica Clinica Aplicada, Amposta, Spain): high-density lipoproteins (HDL), low-density lipoproteins (LDL), liquid triglycerides (TL), lipase, total proteins (TP), and total cholesterol (TC), using a GENESYS 10S UV–Vis spectrophotometer (Thermo scientific, Wisconsin, USA).

### 2.6 Meat fatty acid analysis

After completing the experiment, all guinea pigs in each experimental group were sacrificed at 13 weeks of age by cervical dislocation and cutting of the jugular vein, with exsanguination performed immediately thereafter (40). Tissue samples were obtained from the abdominal muscle, stored at −80 °C for 24 h, and then dried at −81 °C in a Labconco™ FreeZone™ freeze dryer (Fisher Scientific, Madrid, Spain). Total lipid was extracted from 0.2 g of muscle mixed with 50 mL of chloroform-methanol on a magnetic stirrer for 1–2 h. The fatty acid was converted to methyl ester by acetyl chloride; the internal standard was methyl undecanoate (41). Fatty acids were determined by La Molina Calidad Total Laboratorios (Lima, Peru) by gas chromatography coupled to a mass spectrometer (Agilent-7890-MS-5975C, Agilent Technologies, USA), following the guidelines of the bb official method 996.06 of the Association of Official Agricultural Chemists (35).

### 2.7 Intestinal histomorphometry

Tissues were taken from the middle section of the jejunum following the guidelines of Yuan et al. (42) with some modifications. Samples were fixed in 10% formaldehyde for 24 h, dehydrated in ethanol (70–80%–96–100%) for 1 h each, sectioned into 3–5 μm slices, stained with hematoxylin and eosin, and observed with an Olympus optical microscope, BX53F (Tokyo, Japan). In each field, 8–10 villi were selected. The total villi (length and width) and the depths of the Lieberkühn crypts were measured at 10× magnification with the help of cellSens Standard. Ink software. Villus length was measured from the apex of the villus to the apex of the crypt entrance, villus width was measured at the vertical midpoint of the chosen villus, and crypt depth was measured from the crypt entrance to the basal area of the crypt (43).

### 2.8 Metagenomic sequencing and bioinformatic analysis

#### 2.8.1 DNA extraction and library construction

For the extraction of metagenomic DNA collected from the cecal mucosa, the Quick-DNA™ Fungal/Bacterial Miniprep Kit (Zymo Research, USA) was used following the manufacturer’s instructions. DNA quantification and purity were performed using a NanoDrop One Microvolume UV–Vis (Thermo Fisher Scientific, USA) and a Qubit 3 Fluorometer (ThermoFisher Scientific, USA). Paired-end metabarcoding sequencing (2 × 150 bp) of the V3 and V4 hypervariable regions of the bacterial 16S rRNA was performed in a commercial genomic facility, generating approximately 250-bp amplicons.

#### 2.8.2 Bioinformatic analysis

FASTQ files were grouped according to experimental group (T0, T1, and T2). Analysis was performed using the nf-core/Ampliseq v2.9.0 (44) pipeline, run with Nextflow v4.2.1 (45) using the Singularity container. Quality control was performed with FastQC and MultiQC (46, 47). Adapter trimming was performed with Cutadapt (48). Demultiplexing of reads, chimera removal, and amplicon sequence variant (ASV) generation were performed with DADA2 v1.26.0 (49) and QIIME2 (50). ASVs with low sequencing depth were filtered based on rarefaction curves. Taxonomic assignment was performed using the SILVA v138 reference database (51). Sequences identified as chloroplasts, mitochondria, and archaea were removed from the dataset. The rarefaction curve and alpha diversity were assessed via Phyloseq. Modified scripts were performed using the Microeco and MicrobiotaProcess libraries (52, 53). Indices such as Shannon, Pielou’s Uniformity, and the number of OTUs observed were used. Between-group comparisons were performed using the Kruskal–Wallis non-parametric test, applying the Bonferroni correction for multiple comparisons. Beta diversity was assessed by principal coordinate analysis (PCoA) using weighted and unweighted Bray–Curtis, Jaccard, and UniFrac distance metrics, using R v4.4.1. Significant differences were tested by PERMANOVA. Functional taxa and metabolic pathways were analyzed with the differential abundance test (DESeq2) and non-parametric Wilcoxon tests with Bonferroni correction.

### 2.9 Data analysis

Data were analyzed under a completely randomized design with three treatments (T0, T1, and T2). The homogeneity of variances test was performed with Levene’s test (*p* > 0.05). An analysis of variance was performed to determine the significance (*p* < 0.05) and to determine the effects of the diet on the fatty acid profile of the meat, blood parameters, intestinal histomorphometry, productive performance, and traits of sacrifice. The comparison of means was performed with Dunnett’s statistical test (*p* < 0.05), considering T0 as control or without silage inclusion. These data were processed in R v4.3.3 software using the Agricolae library. For principal component analysis, the ggplot2 (54) and Phyloseq (55) libraries were used. The alpha diversity indices (Shannon, Pielou, and Observed Features) were compared using the Kruskal–Wallis test followed by pairwise Wilcoxon rank-sum tests with multiple-test corrections. Beta diversity differences based on Bray–Curtis, Jaccard, and UniFrac distance matrices were evaluated using PERMANOVA (999 permutations). Taxonomic differential abundance was assessed using DESeq2 and non-parametric Wilcoxon tests.

## 3 Results

### 3.1 Meat fatty acid profile

The fatty acid profile of the guinea pig pectoral muscle is presented in Table 2. Inclusion of *O. mykiss* silage significantly (*p* < 0.001) modified most fatty acids, with the exception of heptadecenoic/margaloleic acid, arachidonic acid, and unsaturated fatty acids in *C. porcellus* meat. Diets with silage inclusion resulted in a significant increase (*p* < 0.001) in docosahexaenoic acid (DHA) (C22:6) with respect to T0. Along the same line, total meat fat was also increased at T1 and T2 (*p* < 0.001). Silage supplementation also affected the lipid profile of meat by increasing *ω*-6 and *ω*-9 content (*p* = 0.001, *p* < 0.001, respectively). The only fatty acids that decreased (*p* < 0.001) as silage inclusion in the diets increased were -*γ*- linolenic acid (C18:3 n6), arachidic acid (C20:0), and *ω*-3.

**Table 2:** Fatty acid profile of guinea pig pectoral muscle (g/100 g of muscle).

Fatty acid	T0	T1	T2	*p*-value
Lauric acid (C12:0)	0.058 ± 0.003^b^	0.06 ± 0.003^b^	0.07 ± 0.003^a^	0.006
Myristoleic acid (C14:1)	0.24 ± 0.003^c^	0.27 ± 0.003^b^	0.34 ± 0.003^a^	<0.001
Palmitoleic acid (C16:1)	3.89 ± 0.003^c^	4.27 ± 0.003^b^	4.76 ± 0.018^a^	<0.001
Heptadecanoic/margaric acid (C17:0)	0.12 ± 0.003^b^	0.12 ± 0.003^b^	0.18 ± 0.003^a^	<0.001
Heptadecenoic/margaloleic acid (C17:1)	0.13 ± 0.003^a^	0.12 ± 0.003^b^	0.12 ± 0.003^b^	0.030
Trans-linoleic acid (C18:1 t)	1.22 ± 0.003^c^	1.30 ± 0.003^b^	1.46 ± 0.003^a^	<0.001
Trans-linoleic acid (C18:2 t)	2.62 ± 0.003^b^	2.55 ± 0.003^c^	3.14 ± 0.003^a^	<0.001
-*γ*-linolenic acid (C18:3 n6)	3.52 ± 0.003^a^	3.26 ± 0.003^b^	3.03 ± 0.003^c^	<0.001
Arachidic acid (C20:0)	0.17 ± 0.003^a^	0.16 ± 0.003^b^	0.10 ± 0.003^c^	<0.001
Eicosenoic acid (C20:1 Cis11)	0.00 ± 0.000^b^	0.00 ± 0.000^b^	0.06 ± 0.003^a^	<0.001
Arachidonic acid (C20:4 n6)	0.21 ± 0.003^b^	0.22 ± 0.003^a^	0.21 ± 0.003^b^	0.030
Docosahexaenoic acid (C22:6)	0.00 ± 0.000^c^	0.09 ± 0.003^b^	0.17 ± 0.003^a^	<0.001
Total fat	12.96 ± 0.003^c^	13.25 ± 0.003^b^	14.55 ± 0.003^a^	<0.001
Saturated fats	0.46 ± 0.003^b^	0.45 ± 0.003^c^	0.49 ± 0.003^a^	<0.001
Unsaturated fats	8.08 ± 0.003	8.37 ± 0.003	8.83 ± 0.003	0.252
Monounsaturated fats	4.34 ± 0.003^c^	4.76 ± 0.003^b^	5.35 ± 0.003^a^	<0.001
Polyunsaturated fats	3.84 ± 0.003^a^	3.61 ± 0.003^b^	3.49 ± 0.003^c^	<0.001
Trans fats	3.84 ± 0.003^c^	3.85 ± 0.003^b^	4.59 ± 0.003^a^	<0.001
Trans-11 isomers (18:0 2)	2.62 ± 0.003^b^	2.55 ± 0.003^c^	3.14 ± 0.003^a^	<0.001
Trans-9 isomers (18:0 1)	1.22 ± 0.003^c^	1.30 ± 0.003^b^	1.47 ± 0.003^a^	<0.001
*ω*-3 total	3.52 ± 0.003^a^	3.35 ± 0.003^b^	3.22 ± 0.003^c^	<0.001
*ω*-6 total	0.21 ± 0.003^b^	0.23 ± 0.003^a^	0.23 ± 0.003^a^	0.001
*ω*-9 total	0.06 ± 0.003^b^	0.06 ± 0.003^b^	0.09 ± 0.003^a^	<0.001
*ω*-6/*ω*-3 ratio	0.06 ± 0.002^b^	0.06 ± 0.002^b^	0.07 ± 0.002^a^	<0.001

*γ*, gamma; *ω*, omega.

Mean values ± standard deviation, a,b,c = different letters in each row indicate significant differences at *p* < 0.05.

### 3.2 Blood parameters

#### 3.2.1 Hematological parameters

Table 3 shows the average values of the analysis of hematological variables of the guinea pigs. The total white blood cell count (WBC) presented a decreasing trend from T0 to T2 (*p* = 0.050). The number of lymphocytes (Lym#) was significantly higher at T0 compared to T2 (*p* = 0.037), while the number of monocytes (Mid#) showed no significant difference (*p* = 0.673). On the other hand, red blood cell count (RBC), hemoglobin concentration (HGB), hematocrit (HCT), mean corpuscular volume (MCV), mean corpuscular hemoglobin (MCH), platelets (PLT), and procalcitonin (PCT) remained without significant changes between treatments (*p* > 0.05). Similarly, mean corpuscular hemoglobin concentration (MCHC) decreased significantly from T0 to T2 (*p* < 0.001). In contrast, mean platelet volume (MPV) showed significant differences, being higher at T2 (*p* = 0.020) with respect to the other treatments.

**Table 3:** Hematological parameters of guinea pigs according to treatment.

Hematological variables	T0	T1	T2	*p*-value
WBC (× 10^9^/L)	3.62 ± 1.24^a^	3.46 ± 0.54^a,b^	2.78 ± 0.45^b^	0.050
Lym# (× 10^9^/L)	2.18 ± 0.55^b^	2.66 ± 0.46^a^	2.15 ± 0.47^b^	0.037
Mid# (× 10^9^/L)	0.41 ± 0.20	0.30 ± 0.11	0.44 ± 0.62	0.673
Neu# (× 10^9^/L)	1.03 ± 1.17^a^	0.50 ± 0.24^a,b^	0.38 ± 0.16^b^	0.007
Lym (%)	64.68 ± 20.01	77.00 ± 7.97	74.09 ± 9.25	0.100
RBC (10^12^/L)	4.89 ± 0.35	5.02 ± 0.32	4.96 ± 0.30	0.614
HGB (g/dL)	15.35 ± 0.60	15.24 ± 0.98	14.69 ± 1.10	0.218
HCT (%)	36.24 ± 1.81	37.41 ± 2.15	36.67 ± 2.87	0.479
MCV (fL)	74.31 ± 3.78	74.52 ± 1.98	73.93 ± 2.15	0.879
MCH (pg)	31.50 ± 1.78	36.68 ± 20.93	29.67 ± 1.09	0.385
MCHC (g/dL)	42.38 ± 0.75^a^	40.67 ± 1.05^b^	40.08 ± 0.76^b^	<0.001
PLT (× 10^9^/L)	453.55 ± 102.92	457.36 ± 52.99	427.64 ± 73.34	0.636
MPV (fL)	5.62 ± 0.25^b^	5.94 ± 0.35^a,b^	6.09 ± 0.49^a^	0.020
PCT (%)	0.25 ± 0.06	0.27 ± 0.03	0.27 ± 0.06	0.679

WBC, white blood cell count; Lym#, number of lymphocytes; Mid#, number of monocytes; Neu#, number of granulocytes; Lym%, percentage of lymphocytes; RBC, red blood cell count; HGB, hemoglobin concentration; HCT, hematocrit; MCV, mean corpuscular volume; MCH, mean corpuscular hemoglobin; MCHC, mean corpuscular hemoglobin concentration; PLT, platelet count; MPV, mean platelet volume; PCT, procalcitonin.

Mean values ± standard deviation, a,b = different letters in each row indicate significant differences at *p* < 0.05.

#### 3.2.2 Biochemical parameters

The serum biochemical parameters evaluated in *C. porcellus* showed no significant differences between treatments, with the exception of total cholesterol (TC) (*p* < 0.001) (Table 4). We observed that, when 4% silage was included in the diet, the LC increased with respect to the samples of *C. porcellus* that represented the control group. As for lipoproteins, both high-density lipoproteins (HDL) and low-density lipoproteins (LDL) did not show statistically significant variations between treatments (*p* = 0.374 and *p* = 0.437, respectively), although higher values were observed at T1 for HDL and at T0 for LDL-C. Similarly, liquid triglycerides (TL), lipase, and total proteins remained stable (*p* = 0.913, *p* = 0.256, *p* = 0.263, respectively), with values close to and without relevant changes between treatments.

**Table 4:** Serum biochemical values of guinea pigs according to treatment.

Variables	T0	T1	T2	*p*-value
HDL (mg/dL)	57.43 ± 64.79	91.59 ± 74.90	60.54 ± 42.03	0.374
LDL (mg/dL)	297.89 ± 797.24	43.08 ± 40.37	129.33 ± 118.56	0.437
TL (mg/dL)	99.12 ± 68.22	99.90 ± 46.51	110.28 ± 84.50	0.913
Lipase (U/L)	41.78 ± 25.11	55.46 ± 13.09	46.57 ± 17.73	0.256
TP (g/dL)	4.67 ± 1.47	3.74 ± 1.25	4.32 ± 1.20	0.263
TC (mg/dL)	36.42 ± 8.51^b^	49.51 ± 14.64^b^	81.78 ± 24.68^a^	<0.001

HDL, high-density lipoproteins; LDL, low-density lipoproteins; TL, triglycerides; TP, total proteins; LC, total cholesterol.

Mean values ± standard deviation, a,b = different letters in each row indicate significant differences at *p* < 0.05.

### 3.3 Intestinal histomorphometry

Table 5 shows data on villus height (VH) and width (VW) measurements, as well as crypt depth (DC) and ratio (VH/DC). No significant differences were found in (VH) and (VW), or VH/DC between treatments (*p* > 0.05). However, DC presented significant differences (*p* = 0.047), showing that T2 animals registered a higher value compared to T1, while T0 showed an intermediate value.

**Table 5:** Intestinal histomorphometry of the jejunum.

Variable	T0	T1	T2	*p*-value
VW (μm)	73.63 ± 34.28	71.93 ± 49.41	83.66 ± 20.80	0.723
VH (μm)	510.33 ± 200.33	494.74 ± 236.57	602.70 ± 149.10	0.398
DC (μm)	170.85 ± 55.19^a,b^	124.60 ± 64.26^b^	204.07 ± 91.50^a^	0.047
VH/DC (μm)	3.42 ± 2.26	4.17 ± 2.86	3.23 ± 1.00	0.570

VW, villus width; VH, villus height; DC, depth of the crypt.

Mean values ± standard deviation, a,b = different letters in each row indicate significant differences at *p* < 0.05.

### 3.4 Cecal microbiota

#### 3.4.1 Rarefaction curve

**Figure 1:**
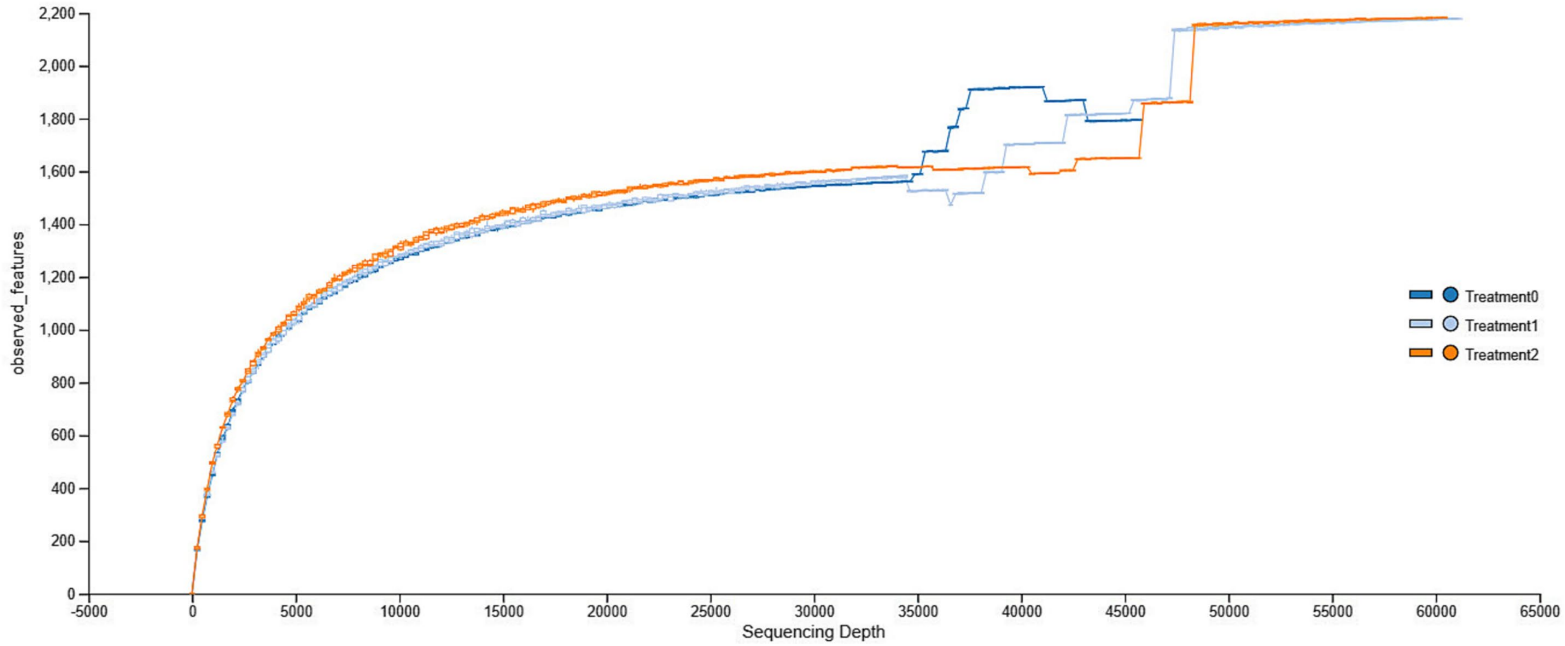
Rarefaction curve of ASVs observed as a function of sequencing depth for Treatment 0, Treatment 1, and Treatment 2 groups.

A total of 3,747,446 raw reads were obtained after sequencing of the V3 and V4 regions of the 16S rRNA gene from 33 samples. Quality filtering was then performed, yielding 2,190,184 clean reads. A total of 22,912 amplicon sequence variants (ASVs) were identified as part of the bacterial community present in guinea pig cecal samples. Rarefaction analysis based on three alpha diversity indices: ACE, Chao1, and Observed Features (observed richness), generated by QIIME2 is shown in Figure 1. The rarefaction curves revealed that the sequencing depth was sufficient to capture the microbial diversity in the guinea pig cecal microbiota, with a plateau reached between 25,000 and 40,000 reads for T0 and T1, where approximately 1,600–1,800 ASVs were observed. In contrast, T2 showed a sustained increase in richness, reaching approximately 2,000 ASV at greater depth, suggesting a greater presence of treatment-influenced taxa (Supplementary Figure S1).

#### 3.4.2 Alpha diversity analysis

**Figure 2:**
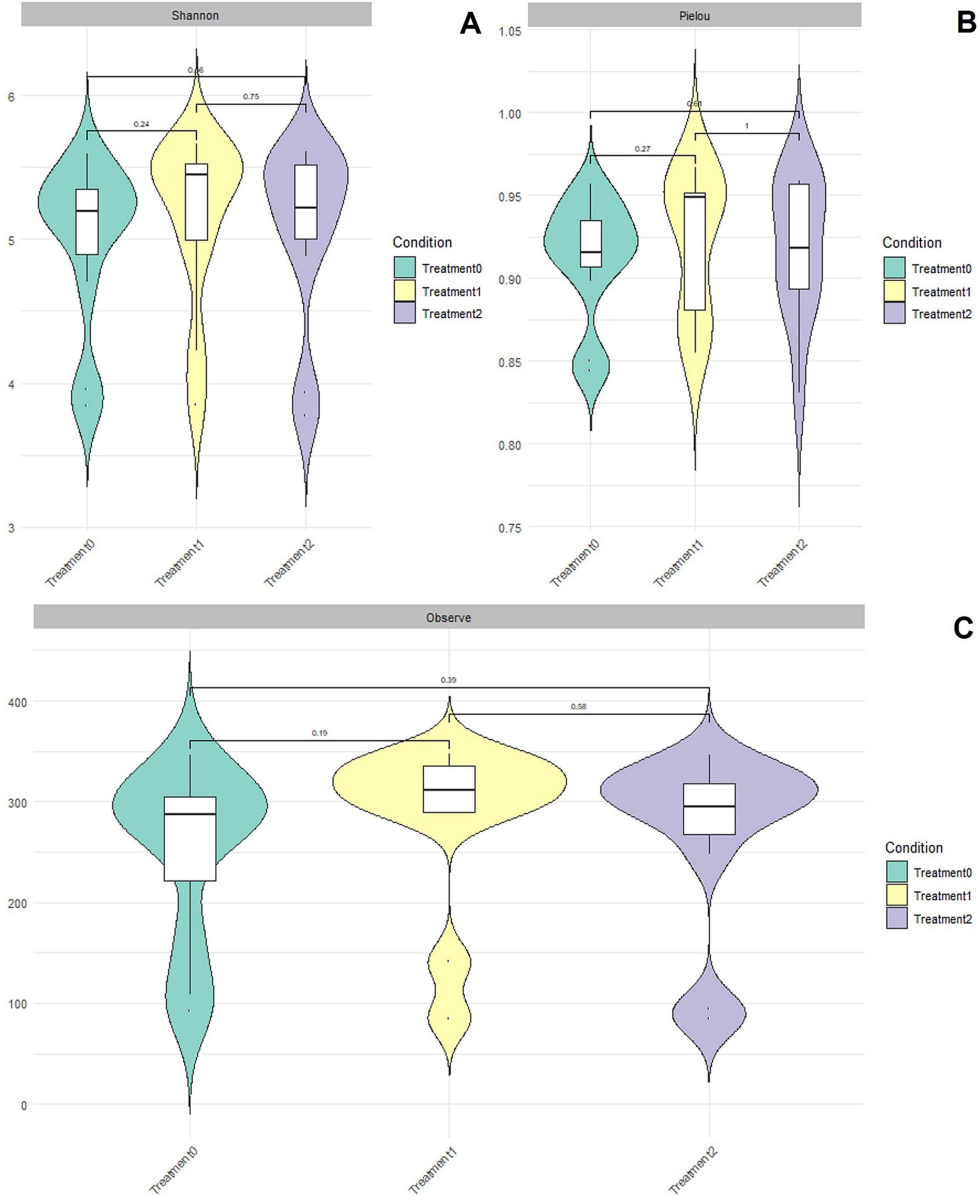
Alpha diversity plots when assessing the richness and abundance of microbial communities using Shannon indices **(A)**, Pielou_Evenness index **(B)**, and Observed Features index **(C)** with significance values (*p* > 0.05) for Treatment 0, Treatment 1, and Treatment 2, using the MicrobiotaProcess library.

The alpha diversity analysis, evaluated by Shannon’s index, showed that the microbial diversity of the cecal microbiota was similar between T0, T1, and T2. The distributions, represented in violin plots, evidenced a slight tendency for the median to increase at T2; however, these differences were not statistically significant (*p* > 0.05) (Figure 2A). The Kruskal–Wallis test confirmed this absence of relevant differences (*H* = 0.265 and *p* = 0.875) (Supplementary Table S1), while paired comparisons also showed no significance (Supplementary Table S2).

The Pielou Evenness index (Figure 2B) indicated an equal distribution of ASV abundances among treatments, with no statistically significant differences (*p* > 0.05). The Kruskal–Wallis test (*H* = 0.317 and *p* = 0.853) (Supplementary Table S3) and paired analyses (*p* = 0.630, 0.630, and 0.895 for T0 to T1, T0 to T2, and T1 to T2 comparisons, respectively) (Supplementary Table S4) confirmed homogeneity between groups, with adjusted *q*-values of 0.895.

The Observed Features index showed that microbial richness was similar between T0 and T1, with a slight non-significant reduction at T2 (*p* > 0.05), possibly associated with the dominance of certain microbial groups (Figure 2C). The Kruskal–Wallis test (*H* = 0.358; *p* = 0.836) (Supplementary Table S5) and adjusted values (*q* = 0.847) confirmed the absence of relevant changes, while paired comparisons also showed no significant differences (*p* = 0.847, 0.564, 0.691 for T0, T1, and T2, respectively) (Supplementary Table S6).

#### 3.4.3 Beta diversity analysis

**Figure 3:**
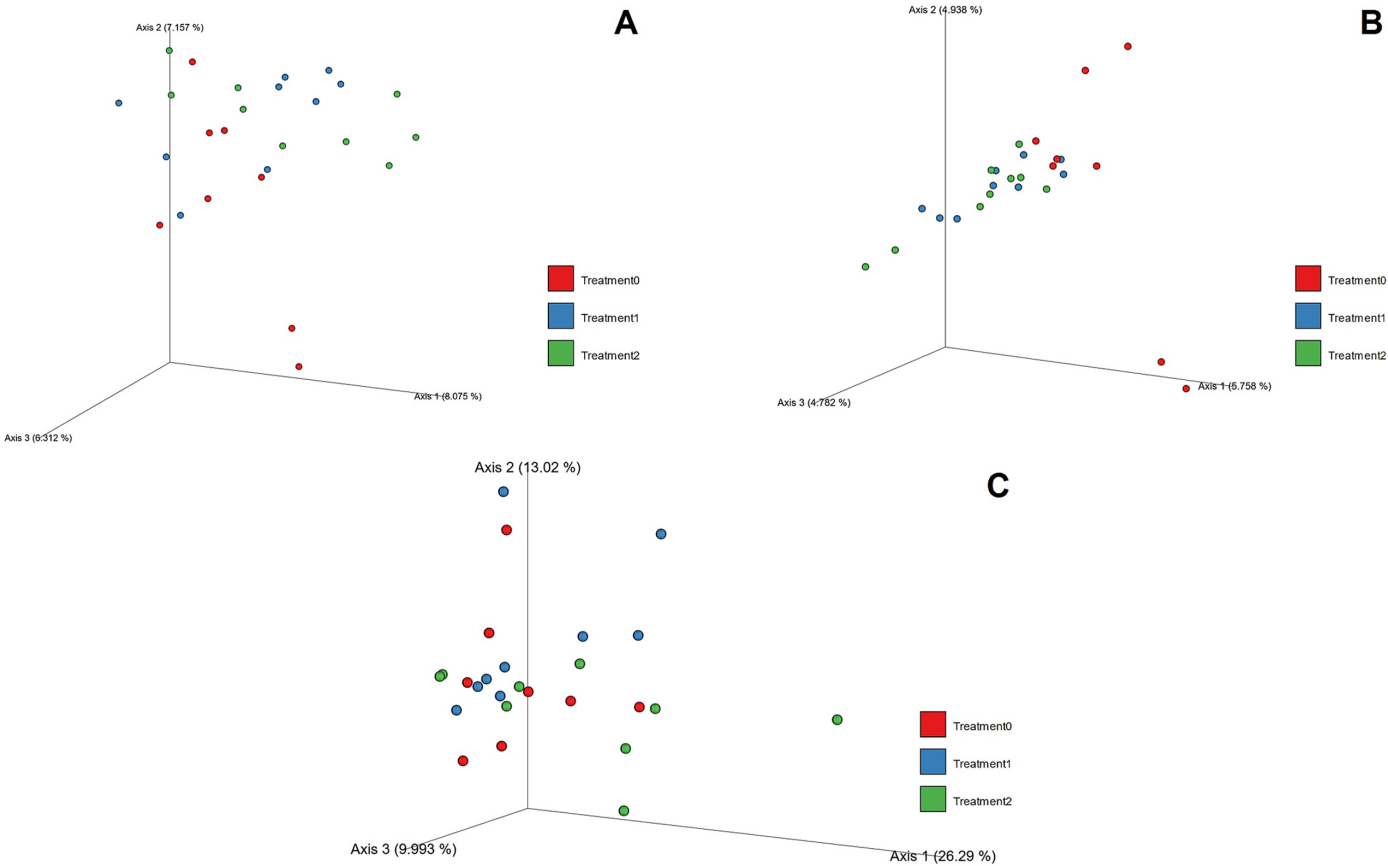
PCoA graph including the Bray–Curtis **(A)**, Jaccard **(B)**, and Weighted UniFrac **(C)** indices for Treatment 0, Treatment 1, and Treatment 2.

The absence of significant changes in the alpha diversity analyses does not imply that the microbial composition was unchanged, as shown in Table 6. Beta diversity analysis, evaluated by Bray–Curtis (Figure 3A), Jaccard (Figure 3B), and Weighted UniFrac distances (Figure 3C), evidenced that the composition of the cecal microbiota did undergo modifications associated with silage consumption. Bray–Curtis (pseudo-*F* = 1.247, *p* = 0.006) and Jaccard (pseudo-*F* = 1.089, *p* = 0.017) metrics showed statistically significant differences between the control group and both silage treatments in both relative abundance and the presence/absence of ASV. In contrast, the Weighted UniFrac distance, which incorporates phylogenetic information, did not detect significant differences (*p* = 0.136).

**Table 6:** PERMANOVA statistical analysis for Bray–Curtis, Jaccard, and Weighted UniFrac distances.

Distances	PERMANOVA results			
	Test statistic name	Test statistic	*p*-value	Number of permutations
Bray–Curtis distance	pseudo-F	1.247	0.006^a^	999
Jaccard distance	pseudo-F	1.089	0.017^a^	999
Weighted UniFrac	pseudo-F	1.297	0.136^b^	999

a,b = different letters in each column indicate significant differences at *p* < 0.05.

Finally, Supplementary Table S7 presents the results of the PERMANOVA analysis applied to the Bray–Curtis, Jaccard, and Weighted UniFrac distances in order to evaluate differences in microbial composition between treatments. The methods based on Bray–Curtis and Jaccard revealed significant differences (*p* < 0.05) between T0 with T1 and T0 with T2, while no differences were observed between T1 with T2 (*p* > 0.05). In contrast, analysis with Weighted UniFrac showed no statistically significant differences between any of the pairs compared, suggesting that the variations detected are mainly associated with differences in the presence/absence and relative abundance of taxa, rather than with the phylogenetic structure of the communities.

#### 3.4.4 Taxonomic composition

**Figure 4:**
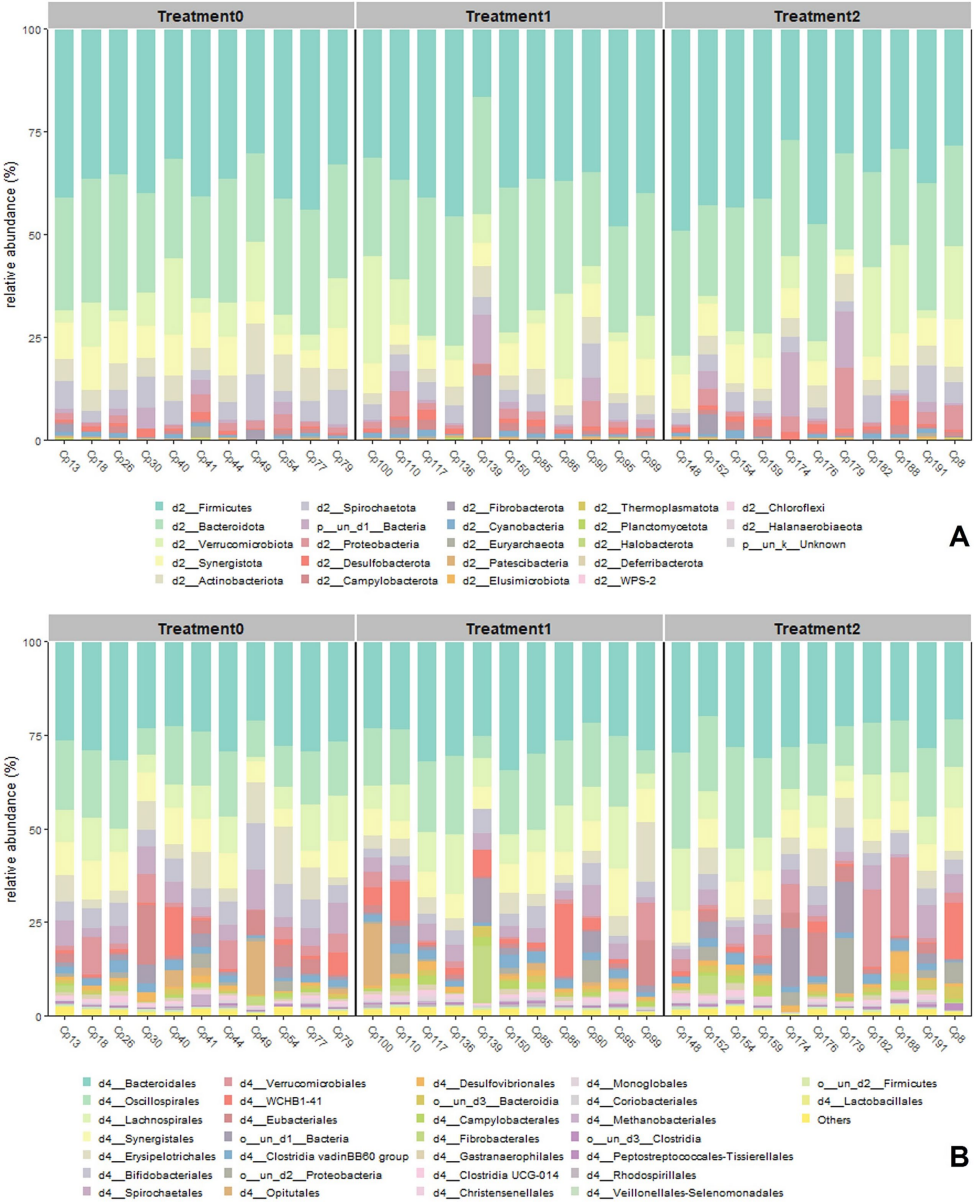
Relative abundance plot of the 30 most abundant phylum **(A)** and 30 most abundant orders **(B)** among the three treatment groups, calculated with the MicrobiotaProcess library.

Analysis of the cecal microbiota showed that, at the Phylum level (Figure 4A), Firmicutes was the dominant group in all treatments (40–60%), followed by Bacteroidetes (30–50%); however, a clear trend was observed in the progressive increase in Bacteroidetes at T0 with respect to T2, while Firmicutes decreased. Other phyla, such as Verrucomicrobiota, Actinobacteria, and Proteobacteria, presented lower abundances (3–10%) (Supplementary Figure S2), but Verrucomicrobiota showed a marked increase at T2.

At the order level (Figure 4B), Bacteroidales and Oscillospirales concentrated the highest proportion of readings, although Bacteroidales increased markedly at T2. This increase was accompanied by an increase in Verrucomicrobiales, Fibrobacterales, and Spirochaetales, all of which are Orders linked to the degradation of structural polysaccharides and adaptations to changes in the protein-rich diet (Supplementary Figure S3).

**Figure 5:**
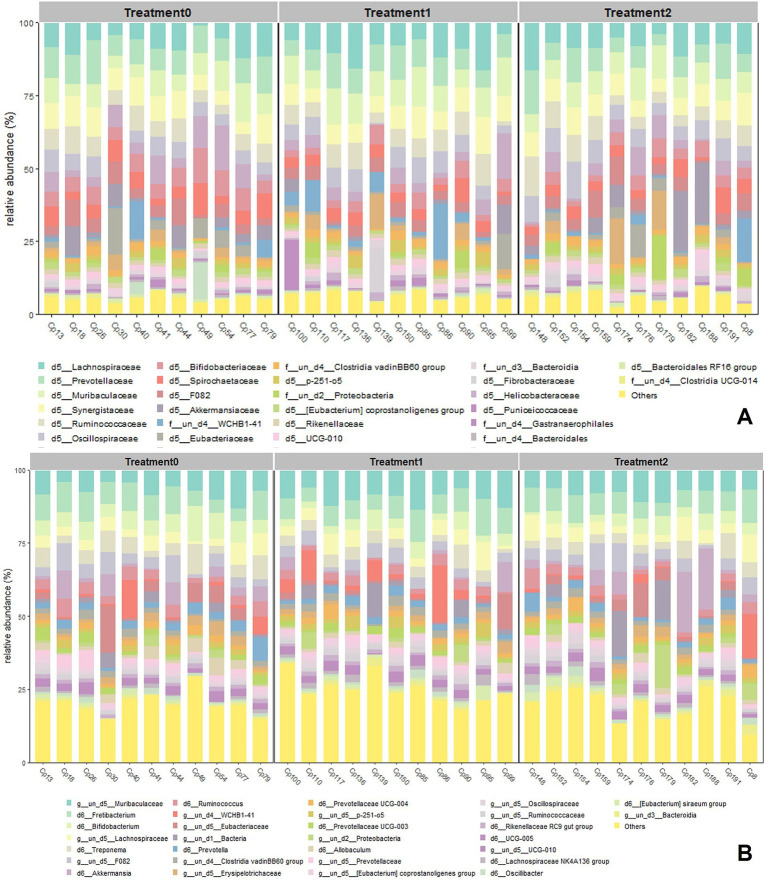
Relative abundance graph of the 30 most abundant family **(A)** and 30 most abundant genus **(B)** among the three treatment groups, calculated with the MicrobiotaProcess library.

At the family level (Figure 5A), Lachnospiraceae and Prevotellaceae were dominant in the three treatments (18–30%, 15–25%, respectively), although Prevotellaceae increased in abundance, especially at T2, as did Synergitaceae and Ruminococcaceae, which also showed notable increases in the same treatment (Supplementary Figure S4).

Genus-level analysis (Supplementary Figure 5B) showed dominance of d5_Muribulaceae with consistent distribution across individuals. Other major genera included Akkermansia, Lachnospiraceae, and Bifidobacterium, with variable proportions, but without markedly differential changes between treatments (Supplementary Figure S5).

**Figure 6:**
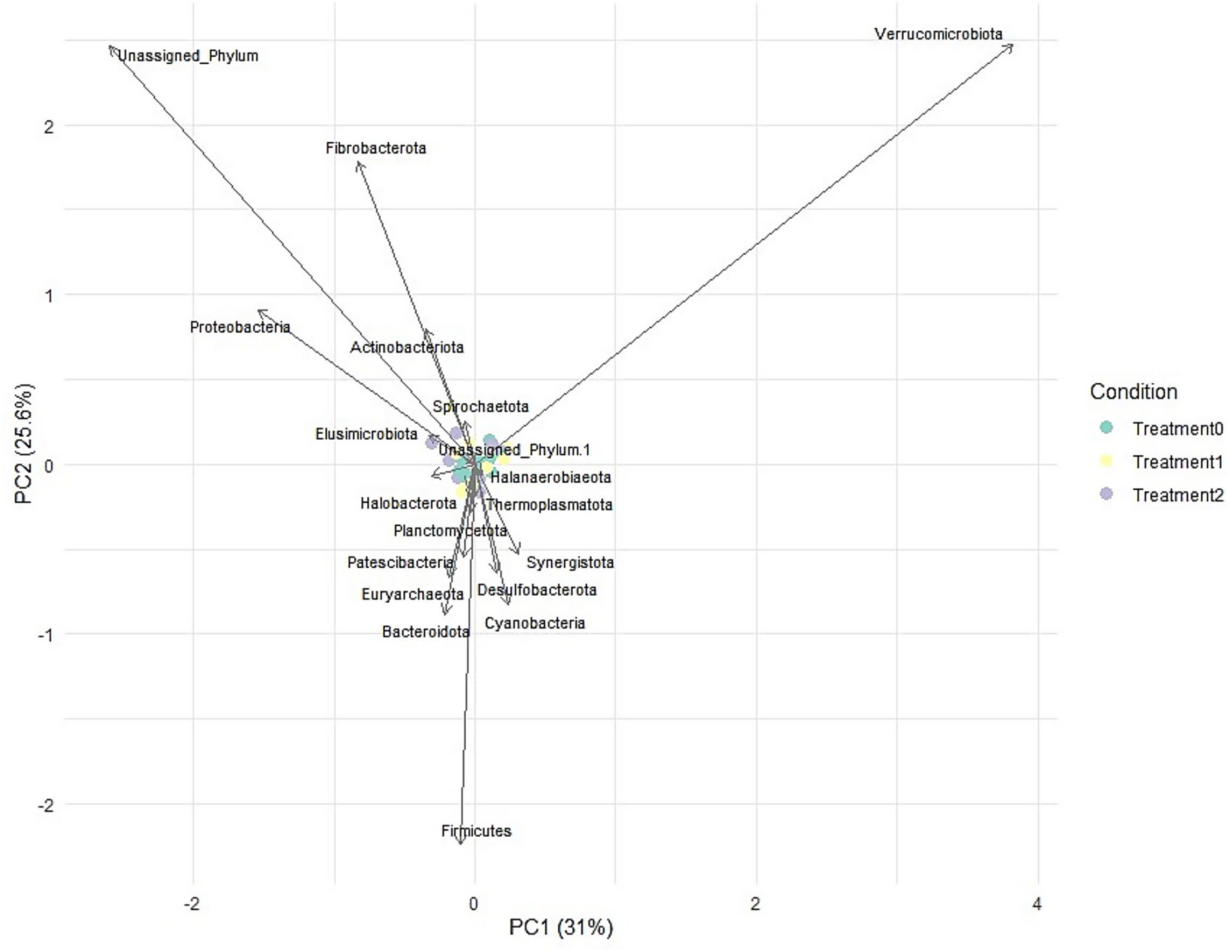
PCA biplot at the phylum level.

Biplot analysis at the phylum level showed that microbial communities did not vary dramatically between treatments, although a slight separation of T2 samples was detected to be associated with the increase in Verrucomicrobia (Figure 6). Most phylum, such as Firmicutes and Bacteroidota, were located near the center of the graph, reflecting their common presence in all groups without significant influence.

Differential abundance analyses (DESeq2) and non-parametric Wilcoxon tests with Bonferroni correction on the 10 most abundant functional taxa confirmed that several bacterial groups and metabolic pathways were significantly enriched or decreased, depending on the treatment (*p* < 0.05) (Supplementary Tables S8, S9).

**Figure 7:**
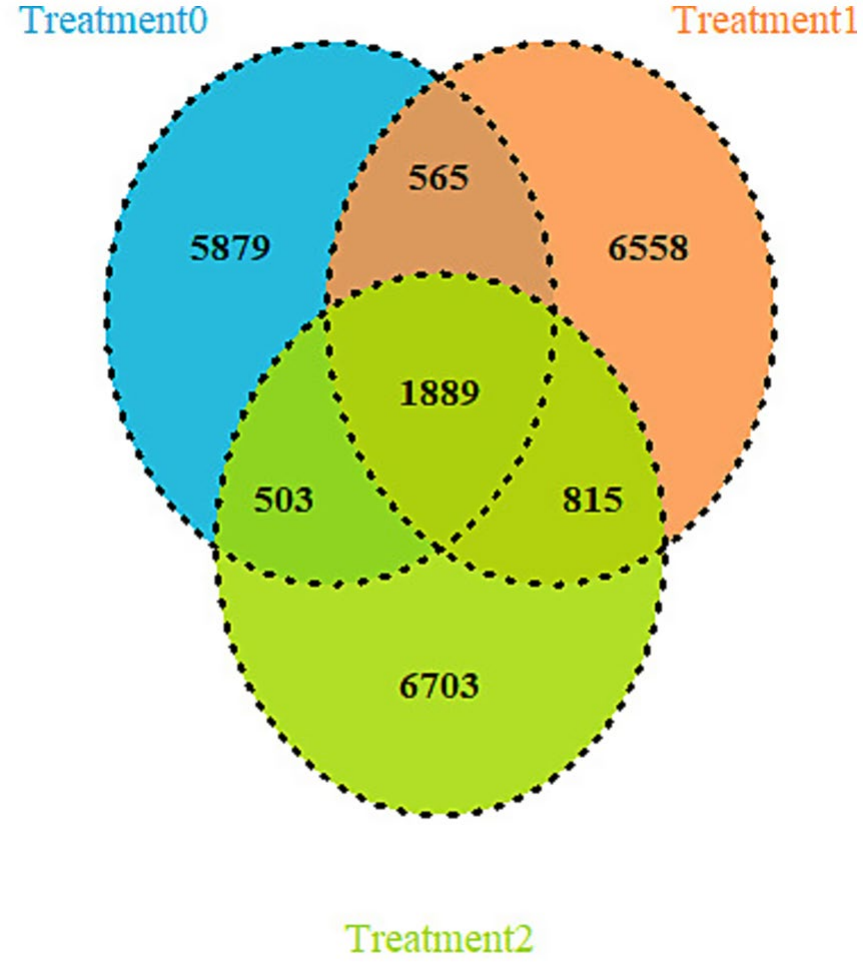
Venn diagram representing the taxa shared between treatments.

Evaluation of the cecal microbial structure revealed distinctive patterns in both the composition and exclusivity of the microbiota. The Venn diagram constructed from the amplicon sequences (ASVs) showed the existence of a conserved microbial core of 1,889 ASVs present in the three treatments (Figure 7).

#### 3.4.5 Functional profile

**Figure 8:**
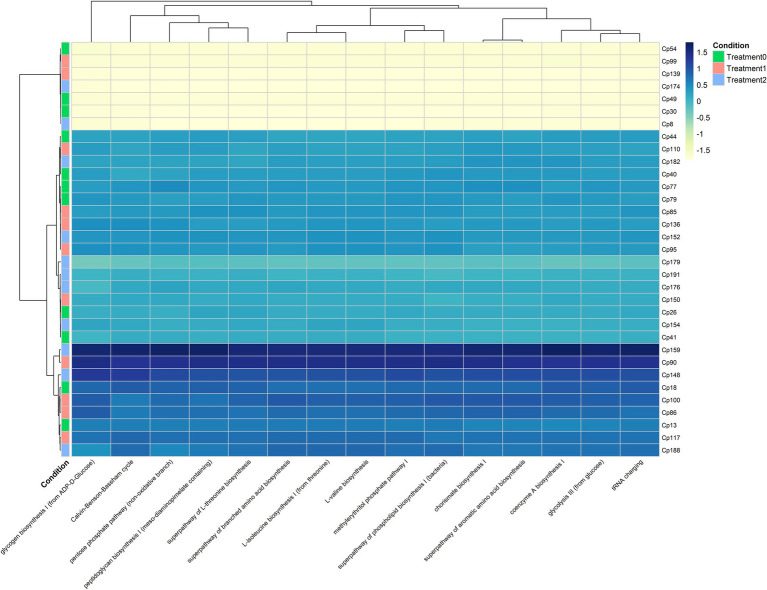
Analysis of the main enzymes involved in the different metabolic processes present in the guinea pig cecum, subjected to treatment with trout silage.

Analysis of the predicted functional profiles of the cecal microbiota showed a remarkably homogeneous distribution among the T0, T1, and T2 groups, with no distinct clustering patterns or abundance gradients suggesting diet-induced alterations (Figure 8). The most representative metabolic pathways included the Calvin–Benson–Bassham cycle, branched-chain amino acid biosynthesis (L-isoleucine, L-valine), glycolysis, oxidative and non-oxidative pentose phosphate pathways, peptidoglycan biosynthesis, and coenzyme A biosynthesis. Hierarchical clustering of ASVs and heat map color intensity confirmed that differences between treatments were minimal and did not show consistent patterns of functional increase or reduction. This interpretation was supported by non-parametric statistical tests (Supplementary Table S10), where all paired comparisons reported *p* > 0.05, indicating no significant differences in the relative abundance of the metabolic pathways evaluated. Taken together, these results suggest that the cecal microbiota maintained functional resilience, preserving its core metabolic repertoire in the face of variations in the level of inclusion of *O. mykiss* silage in the diet.

### 3.5 Productive performance

The productive performance data are presented in Table 7. There were no significant differences (*p* > 0.05) in daily concentrate intake (DCI) or daily pasture intake (DPI) among treatments. However, cumulative weight gain (CWG) was significantly higher in the group with higher silage inclusion T2 compared to the group that did not receive trout silage T0 and T1 (*p* = 0.050). Similarly, the feed conversion factor (FCF) improved significantly (*p* < 0.001) in the animals supplemented with trout silage (T1 and T2), being more efficient in T2.

**Table 7:** Productive performance of guinea pigs according to treatment.

Parameter	T0	T1	T2	*p*-value
DCI (g)	26.24 ± 5.49	26.45 ± 3.20	29.58 ± 3.43	0.126
DPI (g)	114.53 ± 6.48	111.17 ± 6.91	116.58 ± 7.45	0.200
CWG (g)	788.27 ± 110.28^b^	790.18 ± 97.35^b^	884.36 ± 121.26^a^	0.050
FCF (Kg)	4.49 ± 0.22^c^	4.24 ± 0.28^b^	3.94 ± 0.29^a^	<0.001

DCI, daily concentrate intake; DPI, daily pasture intake; CWG, cumulative weight gain; FCF, feed conversion factor.

Mean values ± standard deviation, a,b,c = different letters in each row indicate significant differences at *p* < 0.05.

## 4 Discussion

Underutilized parts of fish such as viscera, skin, and others could be valuable sources of edible proteins and other nitrogenous compounds such as peptides, amino acids, hydrolysates, and lipids, among other bioactive compounds (56). The utilization of these by-products can reduce raw material costs, increase industrial productivity, and minimize environmental pollution (10).

In this study, the inclusion of silage in guinea pig diets modified the lipid profile of the meat. The results suggest that the inclusion of trout silage modifies the lipid profile of the pectoral muscle, increasing mainly monounsaturated fatty acids and certain long-chain fatty acids such as DHA (C22:6). To date, numerous investigations have been conducted to evaluate the possible production of a functional feed by administering diets enriched with *ω*-3 PUFA. For example, Rodriguez et al. (57) evaluated the inclusion of fish oil as a source of fatty acids in rabbit feed, and they found that the total PUFA content and the ratio of *ω*-6/*ω*-3 were lower in the samples of rabbits with enriched diets. This could be primarily due to its content and capacity to transform DHA from fish oil (58). Arachidonic acid (C20:4 *ω*-6) is one of the precursors of prostaglandins and thromboxanes, in addition to regulating body homeostasis (59). DHA (C22:6) is a major constituent of membrane phospholipids in the retina, brain, reproductive tissues, and gametes; therefore, meat from guinea pigs fed trout silage may represent a good source of DHA. The main sources of *ω*-3 PUFA for humans are fish and marine algae, while the requirements of *ω*-6 PUFA are satisfied by meat intake (60). This leads to infer that the production and consumption of PUFA-enriched functional foods, especially guinea pig meat, could be a new complementary strategy to reduce the incidence of several pathologies and improve human health (61). Bouzaida et al. (62) and Scerra et al. (63) found similar results for PUFA *ω*-6 and *ω*-3 in rabbits fed diets containing 20% grape pomace and 10% grape seeds, respectively. The data from our study also show that there was a slight decreasing trend of PUFA *ω*-3, while PUFA *ω*-6 tended to increase. This led to an undesirable increase in the *ω*-6/*ω*-3 ratio; however, this ratio did not even exceed one-fourth of the maximum recommended dietary value of 4 (64). On the other hand, we also found that the content of monounsaturated fatty acids increased as the percentage of silage inclusion in the diets increased. Monounsaturated fatty acids can improve the lipid profile by increasing HDL and reducing LDL and triglycerides (65). There are communities in the world that consume diets rich in monounsaturated fatty acids, such as the Mediterranean. These habits are associated with a lower risk of cardiovascular disease, improved insulin sensitivity, decreased risk of certain types of cancer, and positive effects on cognition, in addition to being considered as one of the world diets for the prevention and treatment of type 2 diabetes (66).

In our research, treatments that had trout silage in the diets modified some of the hematological parameters. Previous studies also indicate that fish silage contains active biopeptides that interfere with the values of different blood parameters (67). Goosen et al (17), who evaluated the inclusion of rainbow trout silage oil in Mozambique tilapia diet and found no differences in the means of hematocrit and red blood cell count, reported similar increases in mean platelet volume values. Although some parameters decreased at higher silage inclusion, their values remained above those reported for native guinea pigs and those raised in humid tropical conditions (68, 69).

Our findings found no significant changes in most of the biochemical values, coinciding with those reported by Ardó et al. (70), who also found no differences when evaluating the incorporation of *Astragalus membranaceus* and *Lonicera japonica* in the serum protein of Nile tilapia. The parameter that did show a statistical difference is total cholesterol, which tends to increase as the incorporation of trout silage in the diet increases. This result differs from that reported by Zamora et al. (71), who found no differences in serum total cholesterol values when increasing levels of sacha inchi oil were incorporated in diets for laying hens. Similarly, Wang et al. (72) found no differences in total cholesterol in pigs. These differences between what was reported are possibly due to the difference in the animal species used in the study and to the different source of food ingredients. No statistical differences were observed in most of the histomorphometric measurements performed, with the exception of crypt depth, where it is shown that, as the inclusion dose of trout silage increases, its depth also increases; this could be due to its properties on the improvement of digestion, absorption, and the functioning of the immune system of animals that consume trout silage (73), besides standing out for having good antioxidant, antithrombotic, antihypertensive, and antibacterial qualities (67).

The guinea pig cecum, a key organ for feed fermentation, harbors a dense and complex microbiota dominated by phyla such as Firmicutes and Bacteroidota, mainly adapted to the digestion of fibrous plant material (74). In this study, the results suggest that the addition of trout silage does not substantially alter the alpha diversity of guinea pig cecal microbiota with respect to the control group. This is consistent with previous observations reporting that high-protein diets promote the proliferation of proteolytic bacteria in the distal gut without substantial changes in microbial composition (75).

Beta diversity analysis indicates that diet reshaped the identity and relative abundance of certain taxa, favoring those better adapted to metabolize the nutrients present in trout silage, possibly with greater proteolytic and lipolytic capacity (75, 76). The lack of significance in the Weighted UniFrac analysis suggests that changes occurred primarily among phylogenetically close taxa, at the species or genus level, within the same bacterial families, without substantial modifications in the larger lineages.

The reduction in Spirochaetaceae between T0 and T1 could be associated with competition for easily fermentable substrates present in the silage, as well as a decrease in intestinal pH, which could affect their growth (77). On the other hand, the increase in Lachnospiraceae and Ruminococcaceae indicates a strengthening of the fermentative capacity of complex fibers and an increased production of short-chain fatty acids (SCFA), particularly butyrate, which is consistent with that reported by Choy et al. (78) and Louis and Flint (79). In addition, the increase in Prevotellaceae suggests that silage treatment may be favoring fiber fermentation, while the reduction in Bacteroidota could signal a shift in microbiome dynamics toward a composition that favors SCFA production.

The Venn diagram indicates a stable community probably composed of generalist taxa well adapted to the basal gut environment (80, 81). However, a high number of exclusive ASVs per treatment was detected, suggesting a marked process of environmental filtering that promoted the formation of specific and specialized ecological niches (82). This phenomenon, in which a dietary intervention significantly alters microbial structure, has been documented in diverse gut ecosystems, where selective pressures modify diversity and favor taxa adapted to particular conditions (83, 84). The greatest intersection of ASV was observed between T1 and T2, indicating that both treatments exerted similar selective pressures, although without fully converging in composition.

Although none of the predicted metabolic pathways showed significant differences between treatments, this study suggests that the cecal microbiota of guinea pigs maintains a highly conserved functional core, regardless of dietary variations. The stability of these pathways is consistent with the concept of functional redundancy, where different taxa can sustain equivalent metabolic functions even when the microbial structure fluctuates (85). The persistence of central pathways such as glycolysis and pentose phosphate is consistent with their universal role in obtaining energy and generating biosynthetic precursors necessary for bacterial proliferation (86). The stability of peptidoglycan biosynthesis and coenzyme A pathways supports the idea that the microbial community maintains essential structural functions necessary for bacterial cell wall and energy metabolism (87, 88). Taken together, these findings indicate that the cecal community of *C. porcellus* responds to the progressive inclusion of *O. mykiss* silage, an important source of bioactive compounds, peptides, vitamins, and DHA, through compositional reorganizations that preserve core functional potential, a pattern of resilience that is physiologically consistent with the fermentative role of the cecum and the need to maintain the production of key metabolites such as short-chain fatty acids (SCFA) for the host.

Regarding productive performance, concentrate intake per day, pasture intake per day, and cumulative weight gain did not show differences. These results differ from those reported by Shabani et al. (89), who did find differences for weight gain values in broilers fed 30, 60, 90, and 120 g/kg levels of fish silage. This could be primarily attributed to the higher protein quality, the biological values of fish silage, and the species under study, although chickens differ from guinea pigs in their digestive system (90). The only parameter that presents statistical difference in our study is the feed conversion factor (FCF), contrary to our results reported (91), who found no differences when evaluating the partial substitution of fish meal for trout viscera silage in red tilapia. Similarly, Vélez and Cadavid (92) reported that the inclusion of red tilapia viscera silage does not affect feed conversion in rabbits, which have a digestive system similar to that of guinea pigs. The positive results for this parameter in our study can be attributed to the balanced nutritional composition of the diets, the high solubility of the silage, and the presence of several fatty acids in the silage (93, 94).

## 5 Conclusion

The findings of this study demonstrate that the inclusion of rainbow trout silage in the guinea pig diet significantly modified the lipid profile of the meat, increasing monounsaturated fatty acids and the DHA content, thereby suggesting its potential for obtaining meat with functional characteristics for human health. On the other hand, the addition of silage did not alter most hematological and biochemical indicators, except for serum cholesterol and some cellular values, which remained within physiological ranges reported for the species. Intestinal histomorphometry showed a greater depth of crypts, which was linked to a better digestive capacity reflected in better feed and immune conversion. Regarding cecal microbiota, although no relevant changes in alpha diversity were evidenced, modifications in beta diversity and taxonomic composition were observed, indicating an adaptive reorganization of the microbial community in the face of silage without compromising its functional stability. From a productive point of view, silage did not significantly affect feed intake or weight gain, but improved the feed conversion factor, reflecting a more efficient use of nutrients. Overall, these results demonstrate that trout silage constitutes a viable alternative ingredient, capable of improving the nutritional quality of meat and favoring sustainability parameters in guinea pig production, without adverse effects on the health and productive performance of the animals.

## Data Availability

The datasets presented in this study can be found in online repositories. The names of the repository/repositories and accession number(s) can be found in the article/Supplementary material.

## References

[1] Avilés DF Martínez AM Landi V Delgado JV. The guinea pig (*Cavia porcellus*): an Andean resource of interest as an agricultural food source. Anim Genet Resour. (2014) 55:87–91. doi: 10.1017/s2078633614000368

[2] de Chauca Zaldívar L., 1997. Guinea pig (*Cavia porcellus*) production. Available online at: https://www.fao.org/4/w6562s/w6562s00.htm#TopOfPage (Accessed June 11, 2025)

[3] Zotte AD Cullere M. Carcass traits and meat quality of rabbit, hare, guinea pig and capybara In: Lorenzo J Munekata P Barba F Toldrá F, editors. More than beef, pork and chicken-the production, processing, and quality traits of other sources of meat for human diet. Cham, Switzerland: Springer International Publishing (2019). 167–210.

[4] MINAGRI (Ministry of Agriculture and Irrigation), 2019. International market potential for guinea pig meat. Directorate of Economic Studies and Agricultural Information. Available online at: https://repositorio.midagri.gob.pe/jspui/handle/20.500.13036/78 (Accessed June 16, 2025)

[5] Reyes-Silva F Aguiar-Novillo S Enríquez-Estrella M Uvidia-Cabadiana H. Analysis of the management, production and commercialization of guinea pig (*Cavia porcellus* L.) in Ecuador. Sci Domain. (2021) 7:1004–18. doi: 10.23857/dc.v7i6.2377

[6] Clemente EJ Arbaiza TF Carcelén FC Lucas OA Bazán VR. Evaluation of the nutritional value of *Puya llatensis* in the feeding of guinea pigs (*Cavia porcellus*). J Vet Res Peru. (2003) 14:1–6. doi: 10.15381/rivep.v14i1.1583

[7] Castro-Bedriñana J Chirinos-Peinado D Quijada-Caro E. Digestible and metabolizable energy prediction models in guinea pig feedstuffs. J Appl Anim Res. (2022) 50:355–62. doi: 10.1080/09712119.2022.2079647

[8] Bardales JAS Segura JLC Robles JLC. Growth of four guinea pig (*Cavia porcellus*) genotypes under two feeding systems. Agric Sci Technol. (2020) 21:e1437. doi: 10.21930/rcta.vol21_num3_art:1437

[9] Yoplac I Yalta J Vásquez HV Maicelo JL. Effect of coffee (*Coffea arabica*) pulp meal as feed on productive parameters of guinea pigs (*Cavia porcellus* L.)-Peru breed. J Vet Res Peru. (2017) 28:549–61. doi: 10.15381/rivep.v28i3.13362

[10] Coppola D Lauritano C Esposito FP Riccio G Rizzo C de Pascale D. Fish waste: from problem to valuable resource. Mar Drugs. (2021) 19:116. doi: 10.3390/MD19020116, 33669858 PMC7923225

[11] Espe M Holen E He J Provan F Chen L Øysæd KB . Hydrolyzed fish proteins reduced activation of caspase-3 in H2O2 induced oxidative stressed liver cells isolated from Atlantic salmon (*Salmo salar*). Springerplus. (2015) 4:658–9. doi: 10.1186/s40064-015-1432-6, 26543792 PMC4628607

[12] Gilbert ER Wong EA Webb KE. Board-invited review: peptide absorption and utilization: implications for animal nutrition and health. J Anim Sci. (2008) 86:2135–55. doi: 10.2527/jas.2007-0826, 18441086

[13] Özyurt G Özkütük AS Uçar Y Durmuş M Ozogul Y. Evaluation of the potential use of discard species for fish silage and assessment of its oils for human consumption. Int J Food Sci Technol. (2019) 54:1081–8. doi: 10.1111/ijfs.13954

[14] Keller M Kreuzer M Reidy B Scheurer A Guggenbühl B Luder M . Effects on performance, carcass and meat quality of replacing maize silage and concentrate by grass silage and corn-cob mix in the diet of growing bulls. Meat Sci. (2022) 188:108795. doi: 10.1016/j.meatsci.2022.108795, 35306298

[15] Raa J Gildberg A Olley JN. Fish silage: a reveiw. Crit Rev Food Sci Nutr. (1982) 16:383–419. doi: 10.1080/10408398209527341, 7047081

[16] Staerfl SM Soliva CR Leiber F Kreuzer M. Fatty acid profile and oxidative stability of the perirenal fat of bulls fattened on grass silage and maize silage supplemented with tannins, garlic, maca and lupines. Meat Sci. (2011) 89:98–104. doi: 10.1016/j.meatsci.2011.04.006, 21550730

[17] Goosen NJ de Wet LF Görgens JF Jacobs K de Bruyn A. Fish silage oil from rainbow trout processing waste as alternative to conventional fish oil in formulated diets for Mozambique tilapia *Oreochromis mossambicus*. Anim Feed Sci Technol. (2014b) 188:74–84. doi: 10.1016/j.anifeedsci.2013.10.019

[18] Goosen NJ de Wet LF Görgens JF. Rainbow trout silage oil as immunity enhancing feed ingredient in formulated diets for South African abalone *Haliotis midae*. Aquaculture. (2014a) 430:28–33. doi: 10.1016/j.aquaculture.2014.03.040

[19] Güllü K Acar Ü Tezel R Yozukmaz A. Replacement of fish meal with fish processing by-product silage in diets for the rainbow trout, *Oncorhynchus mykiss*. Pak J Zool. (2014) 46:1697–703.

[20] Hassaan MS Soltan MA Mohammady EY Elashry MA El-Haroun ER Davies SJ. Growth and physiological responses of Nile tilapia, *Oreochromis niloticus* fed dietary fermented sunflower meal inoculated with *Saccharomyces cerevisiae* and *Bacillus subtilis*. Aquaculture. (2018) 495:592–601. doi: 10.1016/j.aquaculture.2018.06.018

[21] Xie D Ye J Lu M Wang S You C Li Y. Comparsion of activities of fatty acyl desaturases and elongases among six teleosts with different feeding and ecological habits. Front Mar Sci. (2020) 7:117. doi: 10.3389/fmars.2020.00117

[22] Weinroth MD Belk AD Dean C Noyes N Dittoe DK Rothrock MJ . Considerations and best practices in animal science 16S ribosomal RNA gene sequencing microbiome studies. J Anim Sci. (2022) 100:skab346. doi: 10.1093/jas/skab346, 35106579 PMC8807179

[23] Choi NR Na HS Han H Chung J Kim YD. Next-generation sequencing analysis of bacterial species present in the sequestrum of medication-related osteonecrosis of the jaw patients. Arch Oral Biol. (2025) 172:106180–8. doi: 10.1016/j.archoralbio.2025.106180, 39864190

[24] Smart K Pieper JB Viall AK Noxon JO Berger DJ. Comparison of commercial next-generation sequencing assays to conventional culture methods for bacterial identification and antimicrobial susceptibility of samples obtained from clinical cases of canine superficial bacterial folliculitis. Vet Dermatol. (2025) 36:14–23. doi: 10.1111/vde.13299, 39323044 PMC11696477

[25] Forcina G Pérez-Pardal L Carvalheira J Beja-Pereira A. Gut microbiome studies in livestock: achievements, challenges, and perspectives. Animals. (2022) 12:1–19. doi: 10.3390/ani12233375, 36496896 PMC9736591

[26] Hashimoto-Hill S Alenghat T. Inflammation-associated microbiota composition across domestic animals. Front Genet. (2021) 12:649599. doi: 10.3389/fgene.2021.649599, 34239536 PMC8257562

[27] Li X Zheng S Li H Liu J Yang F Zhao X . 16S rRNA sequencing and metabolomics to analyze correlation between fecal flora and metabolites of squabs and parent pigeons. Animals. (2025) 15:1–23. doi: 10.3390/ani15010074, 39795017 PMC11718954

[28] American Veterinary Medical Association. AVMA guidelines for the euthanasia of animals: 2020 ed. Schaumburg, IL, USA: American Veterinary Medical Association (2020).

[29] du Sert NP Ahluwalia A Alam S Avey MT Baker M Browne WJ . Reporting animal research: explanation and elaboration for the arrive guidelines 2.0. PLoS Biol. (2020) 18:e3000411–65. doi: 10.1371/journal.pbio.3000411, 32663221 PMC7360025

[30] Rascón J Angeles WG Huatangari LQ Oliva M Gurbillón MÁB. Dry and wet events in Andean populations of northern Peru: a case study of Chachapoyas, Peru. Front Environ Sci. (2021) 9:614438. doi: 10.3389/fenvs.2021.614438

[31] Chauca-Francia L., 2020. Guinea pig breeding manual. 1st. Lima, Peru: Instituto Nacional de Innovación Agraria (INIA).

[32] Meza E Orellana J Astuhuamán L Mendoza G. Maximization of economic benefits of fattening guinea pig through feed restriction. J Vet Res Peru. (2023) 34:e26374. doi: 10.15381/rivep.v34i5.26374

[33] Toppe J. Olsen R.L. Peñarubia O.R. James D., 2018. Production and utilization of fish silage. A manual on how to turn fish waste into profit and a valuable feed ingredient or fertilizer. Rome, Italy: Food and Agriculture Organization of the United Nations. Available online at: https://openknowledge.fao.org/server/api/core/bitstreams/9b83c384-975b-49f7-9710-745d2cc201a0/content (Accessed March 2, 2024)

[34] AOAC (Association of Official Analytical Chemists). Official methods of analysis. 15th ed. Virginia, USA: AOAC International. (1990).

[35] AOAC (Association of Official Analytical Chemists) 2023 Official method 996.06 fat (Total, saturated, and unsaturated) in foods: hydrolytic extraction gas chromatographic method 22nd ed. New York, USA: Official Methods of Analysis of AOAC International.

[36] Blasco A Ouhayoun J Masoero G. Harmonization of criteria and terminology in rabbit meat research. World Rabbit Sci. (1993) 1:3–10. doi: 10.4995/wrs.1993.189, 39834465

[37] Williams WR Johnston MS Higgins S Izzo AA Kendall LV. Blood profiles in unanesthetized and anesthetized guinea pigs (*Cavia porcellus*). Lab Anim. (2016) 45:35–41. doi: 10.1038/laban.911, 26684957

[38] El-Moghazy M Zedan NS El-Atrsh AM El-Gogary M Tousson E. The possible effect of diets containing fish oil (omega-3) on hematological, biochemical and histopathogical alterations of rabbit liver and kidney. Biomed Prev Nutr. (2014) 4:371–7. doi: 10.1016/j.bionut.2014.03.005

[39] Gaviria YS Londoño LF Zapata JE. Effects of chemical silage of red tilapia viscera (*Oreochromis spp*.) as a source of protein on the productive and hematological parameters in Isa-brown laying hens (*Gallus gallus domesticus*). Heliyon. (2020) 6:e05831. doi: 10.1016/j.heliyon.2020.e05831, 33392405 PMC7773580

[40] El-Gindy YM. The impact of enriching heat-stressed rabbit diets with flaxseed oil with/ without allicin, lycopene, or Punicalagin on antioxidative status, physiological response and meat omega-3. BMC Vet Res. (2025) 21:187. doi: 10.1186/s12917-025-04615-0, 40114098 PMC11924766

[41] Siripongvutikorn S Pumethakul K Thantrirat J Sirinupong N Chansuwan W Usawakesmanee W . Chemical and physical parameters supporting the top-rated product of Kapi-pla, a gastronomy of southern food, Thailand. J Agric Food Res. (2025) 21:101856. doi: 10.1016/j.jafr.2025.101856

[42] Yuan L Zhu C Gu F Zhu M Yao J Zhu C . *Lactobacillus johnsonii* N5 from heat stress-resistant pigs improves gut mucosal immunity and barrier in dextran sodium sulfate-induced colitis. Anim Nutr. (2023) 15:210–24. doi: 10.1016/j.aninu.2023.04.012, 38033603 PMC10685162

[43] Liu Y Fan J Huang H Zhou H Cao Y Zhang Y . High dietary non-starch polysaccharides detrimental to nutrient digestibility, digestive enzyme activity, growth performance, and intestinal morphology in largemouth bass, *Micropterus salmoides*. Front Nutr. (2022) 9:1015371. doi: 10.3389/fnut.2022.1015371, 36386922 PMC9643886

[44] Straub D Blackwell N Langarica-Fuentes A Peltzer A Nahnsen S Kleindienst S. Interpretations of environmental microbial community studies are biased by the selected 16S rRNA (gene) amplicon sequencing pipeline. Front Microbiol. (2020) 11:550420. doi: 10.3389/fmicb.2020.550420, 33193131 PMC7645116

[45] Di Tommaso P Chatzou M Floden EW Barja PP Palumbo E Notredame C. Nextflow enables reproducible computational workflows. Nat Biotechnol. (2017) 35:316–9. doi: 10.1038/nbt.3820, 28398311

[46] Andrews S., 2010. A quality control tool for high throughput sequence data. Available online at: https://www.bioinformatics.babraham.ac.uk/projects/fastqc/ (Accessed June 10, 2025)

[47] Ewels P Magnusson M Lundin S Käller M. MultiQC: summarize analysis results for multiple tools and samples in a single report. Bioinformatics. (2016) 32:3047–8. doi: 10.1093/bioinformatics/btw354, 27312411 PMC5039924

[48] Martin M. Cutadapt removes adapter sequences from high-throughput sequencing reads. EMBnetj. (2011) 17:10–12. doi: 10.14806/ej.17.1.200

[49] Callahan BJ McMurdie PJ Rosen MJ Han AW Johnson AJA Holmes SP. DADA2: high-resolution sample inference from Illumina amplicon data. Nat Methods. (2016) 13:581–3. doi: 10.1038/nmeth.3869, 27214047 PMC4927377

[50] Bolyen E Rideout JR Dillon MR Bokulich NA Abnet CC Al-Ghalith GA . Reproducible, interactive, scalable and extensible microbiome data science using QIIME 2. Nat Biotechnol. (2019) 37:852–7. doi: 10.1038/s41587-019-0209-9, 31341288 PMC7015180

[51] Quast C Pruesse E Yilmaz P Gerken J Schweer T Yarza P . The SILVA ribosomal RNA gene database project: improved data processing and web-based tools. Nucleic Acids Res. (2013) 41:D590–6. doi: 10.1093/nar/gks1219, 23193283 PMC3531112

[52] Liu C Cui Y Li X Yao M. Microeco: an R package for data mining in microbial community ecology. FEMS Microbiol Ecol. (2021) 97:1–9. doi: 10.1093/femsec/fiaa255, 33332530

[53] Xu S Zhan L Tang W Wang Q Dai Z Zhou L . MicrobiotaProcess: a comprehensive R package for deep mining microbiome. The Innovation. (2023) 4:100388. doi: 10.1016/j.xinn.2023.100388, 36895758 PMC9988672

[54] Wickham H. Data Analysis In: Gentleman R Hornik K Parmigiani G, editors. ggplot2: elegant graphics for data analysis. Texas, USA: Springer Cham. (2016). 189–201.

[55] McMurdie PJ Holmes S. Phyloseq: an R package for reproducible interactive analysis and graphics of microbiome census data. PLoS One. (2013) 8:e61217. doi: 10.1371/journal.pone.0061217, 23630581 PMC3632530

[56] Ozogul F Cagalj M Šimat V Ozogul Y Tkaczewska J Hassoun A . Recent developments in valorisation of bioactive ingredients in discard/seafood processing by-products. Trends Food Sci Technol. (2021) 116:559–82. doi: 10.1016/j.tifs.2021.08.007

[57] Rodríguez M Carro MD Valiente V Formoso-Rafferty N Rebollar PG. Effects of dietary fish oil supplementation on performance, meat quality, and cecal fermentation of growing rabbits. J Anim Sci. (2017) 95:3620–30. doi: 10.2527/jas.2017.1690, 28805928

[58] Rizzi L Bochicchio D Bargellini A Parazza P Simioli M. Effects of dietary microalgae, other lipid sources, inorganic selenium and iodine on yolk n-3 fatty acid composition, selenium content and quality of eggs in laying hens. J Sci Food Agric. (2009) 89:1775–81. doi: 10.1002/jsfa.3655

[59] Saini RK Keum YS. Omega-3 and omega-6 polyunsaturated fatty acids: dietary sources, metabolism, and significance-a review. Life Sci. (2018) 203:255–67. doi: 10.1016/j.lfs.2018.04.049, 29715470

[60] Burdge G.C., 2018. Polyunsaturated fatty acid metabolism, 1st. ed. Graham C. B. London, United Kingdom: Elsevier.

[61] Agradi S Sulce M Menchetti L Vigo D Castrica M Barbato O . Dietary supplementation with n-3 polyunsaturated fatty acids: effects on reproductive and productive performance and meat quality in rabbit breeding. Anim Nutr. (2023) 14:70–8. doi: 10.1016/j.aninu.2023.03.009, 37252331 PMC10220468

[62] Bouzaida MD Resconi VC Gimeno D Romero JV Calanche JB Barahona M . Effect of dietary grape pomace on fattening rabbit performance, fatty acid composition, and shelf life of meat. Antioxidants. (2021) 10:795. doi: 10.3390/antiox10050795, 34067887 PMC8155864

[63] Scerra M Foti F Caparra P Bognanno M Fortugno P Viglianti D . Grape seed supplementation in growing rabbits: effect on meat quality. Meat Sci. (2025) 226:109843. doi: 10.1016/j.meatsci.2025.109843, 40334597

[64] Department of Health. Nutritional aspects of cardiovascular disease. London, England: Report on health and social subjects (1994).

[65] Cámara-Martos F Iturbide-Casas M. 13-enteral nutrition formulas: current evidence and nutritional composition. Nutr Beverages. (2019) 12:467–508. doi: 10.1016/B978-0-12-816842-4.00013-7

[66] Schlienger JL Monnier L. Mediterranean diets and diabetes prevention: time for evidence. Metab Dis Med. (2020) 14:626–31. doi: 10.1016/j.mmm.2020.06.020

[67] Daroit DJ Brandelli A. In vivo bioactivities of food protein-derived peptides-a current review. Curr Opin Food Sci. (2021) 39:120–9. doi: 10.1016/j.cofs.2021.01.002

[68] Núñez KPO Coronado PMD Pérez RAA Alfaro-Astorima MI Gómez SB. Hematological reference parameters of indigenous guinea pigs (*Cavia porcellus*). J Vet Res Peru. (2021) 32:e18417. doi: 10.15381/rivep.v32i5.18417

[69] Paredes-López D Robles-Huaynate R Aldava-Pardave U Morales-Cauti M. Changes in the hematology and blood metabolites of guinea pigs (*Cavia porcellus*) under intensive rearing system in humid tropical conditions. La Granja. (2024) 40:130–40. doi: 10.17163/lgr.n40.2024.09

[70] Ardó L Yin G Xu P Váradi L Szigeti G Jeney Z . Chinese herbs (*Astragalus membranaceus* and *Lonicera japonica*) and boron enhance the non-specific immune response of Nile tilapia (*Oreochromis niloticus*) and resistance against *Aeromonas hydrophila*. Aquaculture. (2008) 275:26–33. doi: 10.1016/j.aquaculture.2007.12.022

[71] Zamora SJ Arista MA Fernández PA Valle L Frias H Salvador-Tasayco E . Effect of sacha inchi oil (*Plukenetia volubilis*) on productive performance, egg quality and blood biochemistry of laying hens. J Appl Poult Res. (2025) 34:100537. doi: 10.1016/j.japr.2025.100537

[72] Wang Z Geng C Zhang J Zeng X Wang X Zhang C . Effects of dietary digestible energy levels on growth performance, intestinal function, carcass traits, meat quality and blood biochemical parameters of Ningxiang pigs. Anim Nutr. (2025) 22:522–37. doi: 10.1016/j.aninu.2025.02.012, 40919309 PMC12410524

[73] Gao R Yu Q Shen Y Chu Q Chen G Fen S . Production, bioactive properties, and potential applications of fish protein hydrolysates: developments and challenges. Trends Food Sci Technol. (2021) 110:687–99. doi: 10.1016/j.tifs.2021.02.031

[74] Frias H Valderrama NL Durand GJ Cornejo VG Romani AC Bardales W . Comparative analysis of fasting effects on the cecum microbiome in three guinea pig breeds: Andina, inti, and Peru. Front Microbiol. (2023) 14:1283738. doi: 10.3389/fmicb.2023.1283738, 38173670 PMC10761435

[75] Yao CK Muir JG Gibson PR. Review article: insights into colonic protein fermentation, its modulation and potential health implications. Aliment Pharmacol Ther. (2016) 43:181–96. doi: 10.1111/apt.13456, 26527169

[76] David LA Maurice CF Carmody RN Gootenberg DB Button JE Wolfe BE . Diet rapidly and reproducibly alters the human gut microbiome. Nature. (2014) 505:559–63. doi: 10.1038/nature12820, 24336217 PMC3957428

[77] Weinberg ZG Muck RE. New trends and opportunities in the development and use of inoculants for silage. FEMS Microbiol Rev. (1996) 19:53–68. doi: 10.1016/0168-6445(96)00025-3

[78] Choy WH Adler A Morgan-Lang C Gough EK Hallam SJ Manges AR . Deficient butyrate metabolism in the intestinal microbiome is a potential risk factor for recurrent kidney stone disease. Urolithiasis. (2024) 52:38. doi: 10.1007/s00240-024-01534-x, 38413462

[79] Louis P Flint HJ. Formation of propionate and butyrate by the human colonic microbiota. Environ Microbiol. (2017) 19:29–41. doi: 10.1111/1462-2920.13589, 27928878

[80] Neu AT Allen EE Roy K. Defining and quantifying the core microbiome: challenges and prospects. Proc Natl Acad Sci. (2021) 118:1–10. doi: 10.1073/pnas.2104429118, 34862327 PMC8713806

[81] Sharon I Quijada NM Pasolli E Fabbrini M Vitali F Agamennone V . The core human microbiome: does it exist and how can we find it? A critical review of the concept. Nutrients. (2022) 14:14. doi: 10.3390/nu14142872, 35889831 PMC9323970

[82] Sutton L Mueter FJ Bluhm BA Iken K. Environmental filtering influences functional community assembly of epibenthic communities. Front Mar Sci. (2021) 8:736917. doi: 10.3389/fmars.2021.736917

[83] Nguyen J Lara-Gutiérrez J Stocker R. Environmental fluctuations and their effects on microbial communities, populations and individuals. FEMS Microbiol Rev. (2021) 45:1–16. doi: 10.1093/femsre/fuaa068, 33338228 PMC8371271

[84] Potts LD Douglas A Perez-Calderon LJ Anderson JA Witte U Prosser JI . Chronic environmental perturbation influences microbial community assembly patterns. Environ Sci Technol. (2022) 56:2300–11. doi: 10.1021/acs.est.1c05106, 35103467 PMC9007448

[85] Moya A Ferrer M. Functional redundancy-induced stability of gut microbiota subjected to disturbance. Trends Microbiol. (2016) 24:402–13. doi: 10.1016/j.tim.2016.02.002, 26996765

[86] Ding T Schloss P. Dynamics and associations of microbial community types across the human body. Nature. (2014) 509:357–60. doi: 10.1038/nature13178, 24739969 PMC4139711

[87] Magnúsdóttir S Heinken A Kutt L Ravcheev DA Bauer E Noronha A . Generation of genome-scale metabolic reconstructions for 773 members of the human gut microbiota. Nat Biotechnol. (2017) 35:81–9. doi: 10.1038/nbt.3703, 27893703

[88] Typas A Banzhaf M Gross C Vollmer W. From the regulation of peptidoglycan synthesis to bacterial growth and morphology. Nat Rev Microbiol. (2012) 10:123–36. doi: 10.1038/nrmicro2677, 22203377 PMC5433867

[89] Shabani A Boldaji F Dastar B Ghoorchi T Zerehdaran S Ashayerizadeh A. Evaluation of increasing concentrations of fish waste silage in diets on growth performance, gastrointestinal microbial population, and intestinal morphology of broiler chickens. Anim Feed Sci Technol. (2021) 275:114874. doi: 10.1016/j.anifeedsci.2021.114874

[90] Al-Marzooqi W Al-Farsi MA Kadim IT Mahgoub O Goddard JS. The effect of feeding different levels of sardine fish silage on broiler performance, meat quality and sensory characteristics under closed and open-sided housing systems. Asian Australas J Anim Sci. (2010) 23:1614–25. doi: 10.5713/ajas.2010.10119

[91] Sanguino-Ortiz W Espinosa-Ruiz C Esteban Abad MÁ Román CP Hoyos-Concha JL. Effect of fish meal substitution with trout viscera protein hydrolysate on the innate immune response of red tilapia (*Oreochromis spp*). Fish Physiol Biochem. (2025) 51:56. doi: 10.1007/s10695-024-01444-0, 40011259 PMC11865115

[92] Vélez RA Cadavid SC. Effect of red tilapia (*Oreochromis sp*) waste silage on the productive performance of rabbits (*Oryctolagus cuniculus*). J Vet Res Peru. (2024) 35:e29253. doi: 10.15381/rivep.v35i5.29253

[93] Concha JLH Villada Castillo HS Quintero AF Méndez JRB. Chemical characterization of hydrolyzed protein meal obtained from trout (*Oncorynchus mykiss*) by-products silage. Indian J Sci Technol. (2018) 11:1–13. doi: 10.17485/ijst/2018/v11i16/118634

[94] Perea-Román C Garcés-Caicedo YJ Morales-Barvo YJ Jiménez-Chamorro MA Hoyos-Concha JL Vivas-Quila NJ. Digestibility of enzymatic hydrolysates of animal viscera in *Piaractus brachypomus*, Cuvier 1818. Biotechnol Agric Agroind Sect. (2021) 20:54–67. doi: 10.18684/rbsaa.v20.n1.2022.1606

