# Supplementary material for: Oncorhynchus mykiss silage improves meat fatty acids profile, blood parameters, intestinal histomorphometry, productive performance, and modulates the cecal microbiota of Cavia porcellus
Source: Front Nutr. 2026 Jan 6;12:1725233. doi: 10.3389/fnut.2025.1725233 (PMC12815795; doi:10.3389/fnut.2025.1725233)
Supplement: Supplementary file 1 [file Table_1.DOCX]

**Supplementary data**

| K - W | Result |
| --- | --- |
| H | 0.265 |
| p-value^1^ | 0.875 |

Table S1. Non-parametric Kruskal-Wallis test for all treatments according to the Shannon index.

^1^ = P < 0.05

| Group 1 | Group 2 | H | p-value^1^ | q-value^2^ |
| --- | --- | --- | --- | --- |
| Treatment 0 | Treatment 1 | 0.148 | 0.700 | 0.895 |
| Treatment 0 | Treatment 2 | 0.231 | 0.630 | 0.895 |
| Treatment 1 | Treatment 2 | 0.018 | 0.895 | 0.895 |

Table S2. Paired Kruskal-Wallis nonparametric test for Shannon index.

^1^ = P < 0.05

^2^ = P < 0.01

| K - W | Result |
| --- | --- |
| H | 0.317 |
| p-value^1^ | 0.853 |

Table S3. Non-parametric Kruskal-Wallis test for all treatments according to the Pielou Evenness index.

^1^ = P < 0.05

| Group 1 | Group 2 | H | p-value^1^ | q-value^2^ |
| --- | --- | --- | --- | --- |
| Treatment0 | Treatment1 | 0.231 | 0.630 | 0.895 |
| Treatment0 | Treatment2 | 0.231 | 0.630 | 0.895 |
| Treatment1 | Treatment2 | 0.018 | 0.895 | 0.895 |

Table S4. Paired Kruskal-Wallis non-parametric test for the Pielou Evenness index.

^1^ = P < 0.05

^2^ = P < 0.01

| K - W | Result |
| --- | --- |
| H | 0.358 |
| p-value^1^ | 0.836 |

Table S5. Paired Kruskal-Wallis nonparametric test for Observed Features index.

^1^ = P < 0,05

| Group 1 | Group 2 | H | p-value^1^ | q-value^2^ |
| --- | --- | --- | --- | --- |
| Treatment0 | Treatment1 | 0.037 | 0.847 | 0.847 |
| Treatment0 | Treatment2 | 0.333 | 0.564 | 0.847 |
| Treatment1 | Treatment2 | 0.158 | 0.691 | 0.847 |

Table S6. Paired Kruskal-Wallis nonparametric test for Observed Features index.

^1^ = P < 0.05

^2^ = P < 0.01

Table S7. PERMANOVA of Bray-Curtis, Jaccard Distance and Weighted Unifrac methods.

|  | Comparative treatments | pseudo-F^(¥)^ | p-value^1^ | q-value^2^ |
| --- | --- | --- | --- | --- |
| Bray - Curtis | Treatment 0-Treatment 1 | 1.336**^(¥)^** | 0.008**^(*)^** | 0.015 |
|  | Treatment 0-Treatment 2 | 1.354**^(¥)^** | 0.01**^(*)^** | 0.015 |
|  | Treatment 1-Treatment 2 | 1.073 | 0.193 | 0.193 |
| Jaccard Distance | Treatment 0-Treatment 1 | 1.118**^(¥)^** | 0.018**^(*)^** | 0.027 |
|  | Treatment 0-Treatment 2 | 1.161**^(¥)^** | 0.001**^(*)^** | 0.003**^(**)^** |
|  | Treatment 1-Treatment 2 | 0.996 | 0.536 | 0.536 |
| Weighted UniFrac | Treatment 0-Treatment 1 | 1.183 | 0.304 | 0.304 |
|  | Treatment 0-Treatment 2 | 1.441 | 0.145 | 0.296 |
|  | Treatment 1-Treatment 2 | 1.264 | 0.197 | 0.296 |

^¥^ = higher values represent higher significance

^1^ = P < 0.05

^2^ = P < 0.01

Table S8. Results of differential analysis Deseq2 between T0 and T1.

| **ID ASV** | **baseMean** | **log2FoldChange** | **lfcSE** | **stat** | **p-value** | **padj** | **Kingdom** | **Phylum** | **Class** | **Order** | **Family** |
| --- | --- | --- | --- | --- | --- | --- | --- | --- | --- | --- | --- |
| 3e5b0c6a437c66684e789630be3eca06 | 49.282 | -7.102 | 1.093 | -6.498 | 0.000000 | 0.000000 | Bacteria | Spirochaetota | Spirochaetia | Spirochaetales | Spirochaetaceae |
| 9ac747f740017d56b7927d5e8780b550 | 21.337 | 5.843 | 0.972 | 6.010 | 0.000000 | 0.000004 | Bacteria | Firmicutes | Clostridia | Lachnospirales | Lachnospiraceae |
| 031da83981baaf2fea6330347ffbc323 | 16.794 | 5.195 | 0.910 | 5.710 | 0.000000 | 0.000016 | Bacteria | Firmicutes | Clostridia | Oscillospirales | Ruminococcaceae |
| d05f786ac079d9be73561a9cae9ab5c6 | 50.535 | 5.505 | 1.029 | 5.349 | 0.000000 | 0.000054 | Bacteria | Firmicutes | Clostridia | Lachnospirales | Lachnospiraceae |
| 3b3ae223aae81eef11307af72aca348e | 11.061 | 4.908 | 0.908 | 5.407 | 0.000000 | 0.000054 | Bacteria | Bacteroidota | Bacteroidia | Bacteroidales | Prevotellaceae |
| c79cb1c0ca280fce3ca90571ce3b1fb8 | 49.689 | -5.548 | 1.023 | -5.425 | 0.000000 | 0.000054 | Bacteria | Verrucomicrobiota | Verrucomicrobiae | Verrucomicrobiales | Akkermansiaceae |
| a02ae2d70228de62fa07f0f43d1c6844 | 8.586 | 4.515 | 0.847 | 5.328 | 0.000000 | 0.000054 | Bacteria | Cyanobacteria | Vampirivibrionia | Gastranaerophilales |  |
| af778a0a939a650abc40cbf362590f2a | 10.998 | -4.887 | 0.914 | -5.348 | 0.000000 | 0.000054 | Bacteria | Bacteroidota | Bacteroidia | Bacteroidales | Prevotellaceae |
| 8bc3c1acf34280bce49b63741c515202 | 10.998 | -4.900 | 0.976 | -5.023 | 0.000001 | 0.000247 | Bacteria | Verrucomicrobiota | Verrucomicrobiae | Verrucomicrobiales | Akkermansiaceae |
| e827abcb3179696fe8131e1159bc0abe | 5.109 | 3.593 | 0.729 | 4.929 | 0.000001 | 0.000361 | Bacteria | Desulfobacterota | Desulfovibrionia | Desulfovibrionales | Desulfovibrionaceae |
| 06b1967b046b8582671ca9515a787d8e | 12.283 | 4.771 | 0.975 | 4.892 | 0.000001 | 0.000384 | Bacteria | Firmicutes | Clostridia | Lachnospirales | Lachnospiraceae |
| b8781005587b1d49ba54d6906d3db15f | 22.496 | 4.614 | 0.948 | 4.865 | 0.000001 | 0.000384 | Bacteria | Bacteroidota | Bacteroidia | Bacteroidales | Prevotellaceae |
| fe1929bcc0913757d2c4f38acbd7bc86 | 6.581 | 4.092 | 0.839 | 4.876 | 0.000001 | 0.000384 | Bacteria | Firmicutes | Clostridia | Oscillospirales | Ruminococcaceae |
| da4be2a8937391c65f45724873656f4e | 7.208 | 4.203 | 0.869 | 4.836 | 0.000001 | 0.000412 | Bacteria | Bacteroidota | Bacteroidia | Bacteroidales | Muribaculaceae |
| b66c5cd65597d8d48b4565e2278a7cd3 | 6.675 | -4.116 | 0.860 | -4.785 | 0.000002 | 0.000498 | Bacteria | Verrucomicrobiota | Verrucomicrobiae | Verrucomicrobiales | Akkermansiaceae |
| ff7a5a44de328a9fac5c20825d596912 | 20.836 | -5.018 | 1.054 | -4.759 | 0.000002 | 0.000530 | Bacteria | Synergistota | Synergistia | Synergistales | Synergistaceae |
| 2c2551c0e1787b354dc9224f6cf9c48b | 10.027 | -4.390 | 0.941 | -4.664 | 0.000003 | 0.000676 | Bacteria | Synergistota | Synergistia | Synergistales | Synergistaceae |
| b7283766eb5f7de215ea6514de1ac1e4 | 35.685 | -4.994 | 1.067 | -4.683 | 0.000003 | 0.000676 | Bacteria | Firmicutes | Clostridia | Lachnospirales | Lachnospiraceae |
| 4ed3fc7404d18eee564696b470a0859f | 19.238 | 4.571 | 0.976 | 4.686 | 0.000003 | 0.000676 | Bacteria | Bacteroidota | Bacteroidia | Bacteroidales | Muribaculaceae |
| 066756662796d627e2e242e8840fd963 | 13.943 | 4.327 | 0.928 | 4.665 | 0.000003 | 0.000676 | Bacteria | Bacteroidota | Bacteroidia | Bacteroidales | Prevotellaceae |
| 01a3b1fd4c4a1333ec7ed0da7def8d29 | 6.800 | 3.889 | 0.844 | 4.609 | 0.000004 | 0.000842 | Bacteria | Firmicutes |  |  |  |
| 46613a8b050f0ff86b4f27d5d89d8104 | 8.053 | 4.231 | 0.922 | 4.587 | 0.000004 | 0.000890 | Bacteria | Firmicutes | Clostridia | Lachnospirales | Lachnospiraceae |
| f1e54f8ff749d3c99c149d3a33d74fc5 | 4.795 | -3.572 | 0.783 | -4.565 | 0.000005 | 0.000950 | Bacteria | Bacteroidota | Bacteroidia | Bacteroidales | Muribaculaceae |
| fbc66ddb1338596c3ed97bf0ee674a8d | 5.484 | 3.717 | 0.822 | 4.524 | 0.000006 | 0.001104 | Bacteria | Firmicutes | Clostridia | Oscillospirales | [Eubacterium] coprostanoligenes group |
| adb3fe585c7962745f1795aeb98fafd2 | 5.171 | 3.697 | 0.821 | 4.505 | 0.000007 | 0.001124 | Bacteria | Firmicutes | Clostridia | Oscillospirales | Oscillospiraceae |
| acf08cb2c2051e167ba0c927f0e89888 | 4.513 | -3.470 | 0.770 | -4.503 | 0.000007 | 0.001124 | Bacteria | Bacteroidota | Bacteroidia | Bacteroidales | Prevotellaceae |
| de06d877fe8149feec58b98b682cb0d9 | 5.109 | -3.678 | 0.819 | -4.491 | 0.000007 | 0.001148 | Bacteria | Firmicutes | Clostridia | Clostridia UCG-014 |  |
| 1e4ff3549856ccee1372d6ed9c53bcd8 | 5.737 | 3.804 | 0.853 | 4.462 | 0.000008 | 0.001266 | Bacteria | Bacteroidota | Bacteroidia | Bacteroidales | Muribaculaceae |
| 2e4545398d562535777dd5741d501007 | 4.733 | 3.549 | 0.809 | 4.387 | 0.000011 | 0.001731 | Bacteria | Firmicutes | Clostridia | Oscillospirales | [Eubacterium] coprostanoligenes group |
| 36b06efbf6903d62a00bc1fbad0a61e7 | 5.610 | -3.719 | 0.856 | -4.346 | 0.000014 | 0.002020 | Bacteria | Bacteroidota | Bacteroidia | Bacteroidales | Prevotellaceae |
| d46abfa0f605c126fc7dbbd5d0a405bf | 15.541 | 4.401 | 1.017 | 4.329 | 0.000015 | 0.002084 | Bacteria | Firmicutes | Bacilli | Erysipelotrichales | Erysipelotrichaceae |
| 3758b5e4efe2674edeea6664b4767ea5 | 11.876 | -4.409 | 1.019 | -4.325 | 0.000015 | 0.002084 | Bacteria | Synergistota | Synergistia | Synergistales | Synergistaceae |
| c2f141d351d75c66db17400ef0be237d | 5.798 | -3.887 | 0.903 | -4.305 | 0.000017 | 0.002209 | Bacteria | Verrucomicrobiota | Verrucomicrobiae | Verrucomicrobiales | Akkermansiaceae |
| 32639f00cc49473c7ac97d12a1e64b6e | 19.993 | 4.631 | 1.080 | 4.286 | 0.000018 | 0.002333 | Bacteria | Firmicutes | Clostridia | Oscillospirales | [Eubacterium] coprostanoligenes group |
| 5c67f144fddcad6fa6c52fe969e5aa08 | 3.824 | 3.181 | 0.747 | 4.258 | 0.000021 | 0.002569 | Bacteria | Firmicutes | Clostridia | Oscillospirales | Ruminococcaceae |
| 149d69e53bee1090eecfc69d2aa8c43d | 7.865 | 4.005 | 0.954 | 4.197 | 0.000027 | 0.003288 | Bacteria | Bacteroidota | Bacteroidia | Bacteroidales | Prevotellaceae |
| b3d2c149396f8018780cedae1bb27d68 | 7.051 | 3.930 | 0.940 | 4.180 | 0.000029 | 0.003343 | Bacteria | Bacteroidota | Bacteroidia | Bacteroidales | Prevotellaceae |
| 1f25f124e2ee3737b1a248de980cf83b | 4.137 | 3.319 | 0.793 | 4.183 | 0.000029 | 0.003343 | Bacteria | Bacteroidota | Bacteroidia | Bacteroidales | Muribaculaceae |
| c9e44090e89a88a09291f11792eb0a1f | 8.931 | -3.760 | 0.901 | -4.174 | 0.000030 | 0.003349 | Bacteria | Firmicutes | Clostridia | Oscillospirales | Oscillospiraceae |
| 92fd744ea046a9c7d50402b7d9e6e42a | 10.873 | -3.886 | 0.933 | -4.164 | 0.000031 | 0.003407 | Bacteria | Bacteroidota | Bacteroidia | Bacteroidales | Prevotellaceae |
| 01477f2d820327ef4659292fe0c9fcf1 | 3.699 | 3.123 | 0.757 | 4.125 | 0.000037 | 0.003874 | Bacteria | Bacteroidota | Bacteroidia | Bacteroidales | Muribaculaceae |
| c6dfed6474a6b34b6bdb4c498252d057 | 11.249 | 3.804 | 0.923 | 4.121 | 0.000038 | 0.003874 | Bacteria | Firmicutes | Clostridia | Oscillospirales | Oscillospiraceae |
| 2f0f8bb5a02cf145a3fdb7267147d046 | 4.513 | -3.470 | 0.842 | -4.118 | 0.000038 | 0.003874 | Bacteria | Spirochaetota | Spirochaetia | Spirochaetales | Spirochaetaceae |
| 8413a5ddbe01c683fa9f8c751fb16233 | 5.829 | -3.616 | 0.882 | -4.100 | 0.000041 | 0.004103 | Bacteria | Firmicutes | Clostridia | Oscillospirales | Ruminococcaceae |
| 3c1bdc42aeeba03d7eadf48d80245dbe | 18.768 | 4.357 | 1.066 | 4.086 | 0.000044 | 0.004256 | Bacteria | Firmicutes | Clostridia | Oscillospirales | Ruminococcaceae |
| 6486b1d790134160408f9726835021f2 | 6.550 | 3.109 | 0.765 | 4.061 | 0.000049 | 0.004630 | Bacteria | Actinobacteriota | Coriobacteriia | Coriobacteriales | Atopobiaceae |
| 5ea41fe95a274cb202feec290ab190cb | 3.761 | 3.152 | 0.777 | 4.055 | 0.000050 | 0.004665 | Bacteria | Firmicutes | Clostridia | Oscillospirales | Ruminococcaceae |
| 64ce0486c2676ab1c48ebea626ebbb19 | 3.855 | -3.197 | 0.795 | -4.020 | 0.000058 | 0.005299 | Bacteria | Desulfobacterota | Desulfovibrionia | Desulfovibrionales | Desulfovibrionaceae |
| 4f7ba303e98cdc9c5e33b6790ed5b36b | 4.043 | -3.280 | 0.817 | -4.014 | 0.000060 | 0.005312 | Bacteria | Firmicutes | Clostridia | Oscillospirales | Butyricicoccaceae |
| 08a41e01de0abe3c4728a555128525b2 | 3.824 | 3.138 | 0.786 | 3.993 | 0.000065 | 0.005602 | Bacteria | Firmicutes | Bacilli | Izemoplasmatales |  |
| 64304cb0171f363fd1822cfab4fc153d | 3.981 | -3.253 | 0.815 | -3.992 | 0.000065 | 0.005602 | Bacteria | Bacteroidota | Bacteroidia | Bacteroidales | p-251-o5 |
| e5b567775de40ee2fda74d67d3b5e399 | 7.082 | 3.238 | 0.813 | 3.982 | 0.000068 | 0.005739 | Bacteria | Firmicutes | Clostridia | Oscillospirales | Oscillospiraceae |
| 668abbcf5605bf57429af296621cb4c4 | 3.730 | 3.138 | 0.789 | 3.976 | 0.000070 | 0.005763 | Bacteria | Firmicutes | Clostridia | Lachnospirales | Lachnospiraceae |
| e56f83b42f85d652f589de8fb9ac0ff1 | 4.764 | 3.370 | 0.849 | 3.968 | 0.000072 | 0.005850 | Bacteria | Firmicutes | Clostridia | Oscillospirales | Ruminococcaceae |
| 68060cf41d3d5c75475cb5e68a5d8b0c | 5.077 | -3.446 | 0.870 | -3.960 | 0.000075 | 0.005944 | Bacteria | Actinobacteriota | Actinobacteria | Bifidobacteriales | Bifidobacteriaceae |
| bb691ff57450e8fc41e733092563dcb8 | 14.194 | 4.076 | 1.034 | 3.942 | 0.000081 | 0.006301 | Bacteria | Bacteroidota | Bacteroidia | Bacteroidales | F082 |
| ba986a10fd2d8dd2edd7d85eb7910291 | 4.607 | 3.167 | 0.811 | 3.904 | 0.000094 | 0.006866 | Bacteria | Firmicutes | Clostridia | Oscillospirales | Oscillospiraceae |
| c154ae336d35b0265eea8634438c208a | 3.385 | -2.965 | 0.760 | -3.901 | 0.000096 | 0.006866 | Bacteria | Firmicutes | Clostridia | Oscillospirales | Oscillospiraceae |
| 8ccbd5d2f7d9a978496ed3a2fc6ba191 | 6.236 | 3.265 | 0.838 | 3.897 | 0.000097 | 0.006866 | Bacteria | Firmicutes | Clostridia | Oscillospirales | UCG-010 |
| 34d93ae30156e7288142fd615dc21443 | 6.738 | 3.677 | 0.942 | 3.904 | 0.000094 | 0.006866 | Bacteria | Campylobacterota | Campylobacteria | Campylobacterales | Helicobacteraceae |
| 3e77c8a8a083d91dfa69a8ef33b92f03 | 6.769 | 3.625 | 0.930 | 3.899 | 0.000097 | 0.006866 | Bacteria | Firmicutes | Clostridia | Clostridia vadinBB60 group |  |
| 61ea227a617e6edd71bd97f5b163a41d | 3.761 | -3.154 | 0.806 | -3.911 | 0.000092 | 0.006866 | Bacteria | Firmicutes | Clostridia | Oscillospirales | Oscillospiraceae |
| 6ef87a3435743b5bcbab03d485b8be17 | 3.981 | -3.114 | 0.804 | -3.872 | 0.000108 | 0.007483 | Bacteria | Proteobacteria | Gammaproteobacteria | Coxiellales | Coxiellaceae |
| 4c386b2df447db461846104a379fb96c | 3.135 | -2.825 | 0.731 | -3.867 | 0.000110 | 0.007530 | Bacteria | Synergistota | Synergistia | Synergistales | Synergistaceae |
| 20b15799d8559e451b3193163148c5d3 | 3.135 | -2.825 | 0.732 | -3.857 | 0.000115 | 0.007702 | Bacteria | Firmicutes | Clostridia | Lachnospirales | Lachnospiraceae |
| 87e3a56d1f678f7811394b8ea204280b | 4.043 | 3.137 | 0.815 | 3.851 | 0.000117 | 0.007773 | Bacteria | Bacteroidota | Bacteroidia | Bacteroidales | Muribaculaceae |
| c1c57e283677085e4347bb97be5ead3a | 2.980 | -2.730 | 0.713 | -3.829 | 0.000128 | 0.008371 | Bacteria | Bacteroidota | Bacteroidia | Bacteroidales | Muribaculaceae |
| 823fbd932e7d9185e494e22e4e11e58a | 3.542 | 3.045 | 0.797 | 3.820 | 0.000133 | 0.008570 | Bacteria | Firmicutes | Clostridia | Lachnospirales | Lachnospiraceae |
| 2f3b74374566615ec1e43ce49c05a972 | 6.236 | 3.370 | 0.884 | 3.812 | 0.000138 | 0.008723 | Bacteria | Firmicutes | Clostridia | Lachnospirales | Lachnospiraceae |
| 383c0ab19cfc0b051736cd571fa24663 | 10.654 | 3.877 | 1.019 | 3.807 | 0.000141 | 0.008789 | Bacteria | Spirochaetota | Spirochaetia | Spirochaetales | Spirochaetaceae |
| d413e306752775b7bfe2cc78d4059502 | 3.166 | -2.843 | 0.748 | -3.802 | 0.000144 | 0.008844 | Bacteria | Firmicutes | Clostridia | Clostridia vadinBB60 group |  |
| f749bb08534f7dbdeaca5938155f783f | 3.229 | -2.879 | 0.759 | -3.794 | 0.000148 | 0.008875 | Bacteria | Bacteroidota | Bacteroidia | Bacteroidales | Prevotellaceae |
| c5137a60a24ab33d591d0d5dc06774a1 | 98.844 | -3.816 | 1.005 | -3.795 | 0.000148 | 0.008875 | Bacteria | Bacteroidota | Bacteroidia | Bacteroidales | Prevotellaceae |
| b7bbacd6c95cfb31bfa7da256e600f38 | 3.448 | -2.998 | 0.793 | -3.782 | 0.000155 | 0.009172 | Bacteria | Bacteroidota | Bacteroidia | Bacteroidales | Muribaculaceae |
| 43b22a5c6ddbedde7794a73ec50bd278 | 3.135 | 2.823 | 0.747 | 3.778 | 0.000158 | 0.009204 | Bacteria | Firmicutes | Clostridia | Oscillospirales | Ruminococcaceae |
| 72e16576608690ae25c7fdacc4eda705 | 20.930 | 3.060 | 0.811 | 3.774 | 0.000161 | 0.009232 | Bacteria | Firmicutes | Clostridia | Oscillospirales | Oscillospiraceae |
| 5aa7eee9c1dc57ced5e5b5e436f1693a | 3.041 | 2.767 | 0.742 | 3.728 | 0.000193 | 0.009919 | Bacteria | Bacteroidota | Bacteroidia | Bacteroidales | Muribaculaceae |
| a9b0696a24529feab8ff6fbd3045f518 | 8.962 | 2.738 | 0.733 | 3.734 | 0.000188 | 0.009919 | Bacteria | Bacteroidota | Bacteroidia | Bacteroidales | Muribaculaceae |
| 1fda4849891baee7935980ef0a3adacd | 3.072 | 2.786 | 0.745 | 3.741 | 0.000184 | 0.009919 | Bacteria | Bacteroidota | Bacteroidia | Bacteroidales | Muribaculaceae |
| 97cfa655d085e9be6f368e865314877e | 7.396 | 3.408 | 0.914 | 3.729 | 0.000192 | 0.009919 | Bacteria | Desulfobacterota | Desulfovibrionia | Desulfovibrionales | Desulfovibrionaceae |
| be4b54c0530ce5f658a72f14d567f60f | 3.448 | -2.897 | 0.774 | -3.744 | 0.000181 | 0.009919 | Bacteria | Bacteroidota | Bacteroidia |  |  |
| 69c6251e78f702924ddfcc618f5fcb47 | 11.124 | 3.614 | 0.964 | 3.749 | 0.000178 | 0.009919 | Bacteria | Firmicutes | Clostridia | Oscillospirales | Oscillospiraceae |
| a6a05edbfa364be7c1a70e70dd8d64ab | 3.166 | -2.843 | 0.760 | -3.739 | 0.000184 | 0.009919 | Bacteria | Synergistota | Synergistia | Synergistales | Synergistaceae |
| 69900980a6e4f5c456a5617d098e496c | 3.041 | -2.768 | 0.743 | -3.728 | 0.000193 | 0.009919 | Bacteria | Firmicutes | Clostridia | Oscillospirales | Oscillospiraceae |
| d3dacdcf8cb2e32cb0b60a887c5ba7ae | 3.354 | -2.948 | 0.788 | -3.740 | 0.000184 | 0.009919 | Bacteria | Bacteroidota | Bacteroidia | Bacteroidales | p-251-o5 |
| c33f5eaff1fe26966859a938f7ff15d5 | 2.790 | -2.605 | 0.701 | -3.718 | 0.000201 | 0.010200 | Bacteria | Firmicutes | Clostridia | Christensenellales | Christensenellaceae |
| 81fb62f62c2076bfab4effd143b62f9b | 3.041 | 2.767 | 0.747 | 3.706 | 0.000211 | 0.010568 | Bacteria | Bacteroidota | Bacteroidia | Bacteroidales | Tannerellaceae |
| f813eee0d7c1ea5b0086954a86ca259b | 3.072 | -2.787 | 0.754 | -3.698 | 0.000218 | 0.010794 | Bacteria | Bacteroidota | Bacteroidia | Bacteroidales | Rikenellaceae |
| 8480c239df2ab729c748663dd1e1760b | 6.424 | -3.583 | 0.974 | -3.680 | 0.000233 | 0.011453 | Bacteria | Firmicutes | Clostridia | Oscillospirales | Ruminococcaceae |
| d5a75129aa3f9480fb8a0c8756a5c494 | 4.701 | 3.196 | 0.871 | 3.671 | 0.000242 | 0.011727 | Bacteria | Firmicutes | Clostridia | Oscillospirales | Oscillospiraceae |
| c8bd81a949c32dfc0cee47c42d31a951 | 3.479 | 2.823 | 0.772 | 3.657 | 0.000256 | 0.012268 | Bacteria | Bacteroidota | Bacteroidia |  |  |
| e42215c60c65378e53cc9d52408b2eef | 3.166 | 2.841 | 0.779 | 3.649 | 0.000263 | 0.012488 | Bacteria | Desulfobacterota | Desulfuromonadia | Bradymonadales |  |
| 4eb71124b24d0d18e77b3d693b29b402 | 6.037 | -2.432 | 0.667 | -3.644 | 0.000269 | 0.012488 | Bacteria | Synergistota | Synergistia | Synergistales | Synergistaceae |
| 4e60d69076dd70139ea71351716711fd | 7.991 | 3.456 | 0.949 | 3.644 | 0.000269 | 0.012488 | Bacteria | Verrucomicrobiota | Verrucomicrobiae | Verrucomicrobiales | Akkermansiaceae |
| 3d850ab169b8ecc68fb9102c17c6bccb | 4.419 | 3.123 | 0.858 | 3.640 | 0.000273 | 0.012529 | Bacteria | Firmicutes | Clostridia | Oscillospirales | Oscillospiraceae |
| 98e7122a698d16adaff4ad72779958bc | 4.106 | -2.736 | 0.753 | -3.631 | 0.000282 | 0.012822 | Bacteria | Bacteroidota | Bacteroidia | Bacteroidales | Prevotellaceae |
| 681cb9ec1e3cc99f61b6ea54a3c6b6fd | 5.359 | 3.123 | 0.861 | 3.628 | 0.000285 | 0.012843 | Bacteria | Firmicutes | Clostridia | Lachnospirales | Lachnospiraceae |
| f0e6fe0ce95507e68fee9adf17d69a0b | 3.103 | 2.804 | 0.775 | 3.617 | 0.000297 | 0.013255 | Bacteria | Firmicutes | Clostridia | Oscillospirales | Ruminococcaceae |
| 5531e351042d1bc2e5acc59cb4260d69 | 24.376 | 3.719 | 1.029 | 3.613 | 0.000302 | 0.013338 | Bacteria | Firmicutes | Clostridia | Oscillospirales | Ruminococcaceae |

Table S9. Results of differential analysis Deseq2 between T0 and T2.

| **ID ASV** | **baseMean** | **log2FoldChange** | **lfcSE** | **stat** | **p-value** | **padj** | **Kingdom** | **Phylum** | **Class** | **Order** | **Family** |
| --- | --- | --- | --- | --- | --- | --- | --- | --- | --- | --- | --- |
| 3ac534a247bac69fc0c67c1614118f3d | 18.047 | 5.338 | 0.986 | 5.416 | 6.10116E-08 | 0.00026 | Bacteria | Spirochaetota | Spirochaetia | Spirochaetales | Spirochaetaceae |
| 72e16576608690ae25c7fdacc4eda705 | 20.930 | 4.298 | 0.807 | 5.327 | 9.99341E-08 | 0.00026 | Bacteria | Firmicutes | Clostridia | Oscillospirales | Oscillospiraceae |
| e92faee5d0fa500988e971c5d686a812 | 15.008 | 5.126 | 0.994 | 5.155 | 2.53685E-07 | 0.00026 | Bacteria | Synergistota | Synergistia | Synergistales | Synergistaceae |
| bf238934786ca90893a770b53a59b8e6 | 8.931 | 4.576 | 0.883 | 5.184 | 2.17436E-07 | 0.00026 | Bacteria | Spirochaetota | Spirochaetia | Spirochaetales | Spirochaetaceae |
| e0347bb1dcb22549b65cc8e49c01fa1d | 16.105 | -5.287 | 1.013 | -5.219 | 1.79716E-07 | 0.00026 | Bacteria | Synergistota | Synergistia | Synergistales | Synergistaceae |
| 62a379b0dd1c179302ef44929399cf84 | 6.236 | 4.005 | 0.787 | 5.088 | 3.62418E-07 | 0.00027 | Bacteria | Firmicutes | Clostridia | Lachnospirales | Lachnospiraceae |
| af778a0a939a650abc40cbf362590f2a | 10.998 | -4.540 | 0.891 | -5.092 | 3.53873E-07 | 0.00027 | Bacteria | Bacteroidota | Bacteroidia | Bacteroidales | Prevotellaceae |
| 8f3e4eef53e7b03aa8a0e5f484372bb7 | 13.035 | -5.006 | 0.993 | -5.041 | 4.63894E-07 | 0.00029 | Bacteria | Firmicutes | Clostridia | Oscillospirales | Ruminococcaceae |
| 8bc3c1acf34280bce49b63741c515202 | 10.998 | -4.900 | 0.976 | -5.023 | 5.09746E-07 | 0.00029 | Bacteria | Verrucomicrobiota | Verrucomicrobiae | Verrucomicrobiales | Akkermansiaceae |
| c5137a60a24ab33d591d0d5dc06774a1 | 98.844 | -4.942 | 1.011 | -4.886 | 1.0296E-06 | 0.00048 | Bacteria | Bacteroidota | Bacteroidia | Bacteroidales | Prevotellaceae |
| 841a16f45b755c53388bd66ac3d0cc71 | 11.030 | 4.002 | 0.819 | 4.887 | 1.02352E-06 | 0.00048 | Bacteria | Bacteroidota | Bacteroidia | Bacteroidales | Prevotellaceae |
| 3e5b0c6a437c66684e789630be3eca06 | 49.282 | -4.979 | 1.024 | -4.861 | 1.1686E-06 | 0.00050 | Bacteria | Spirochaetota | Spirochaetia | Spirochaetales | Spirochaetaceae |
| ab680950a9f4988c8bbea05fb2caf7ef | 10.184 | 4.587 | 0.947 | 4.843 | 1.27594E-06 | 0.00050 | Bacteria | Firmicutes | Clostridia | Oscillospirales | Butyricicoccaceae |
| acb3d262d60930bdd67cc2fc40357d7c | 10.058 | 4.286 | 0.887 | 4.831 | 1.35919E-06 | 0.00050 | Bacteria | Firmicutes | Clostridia | Oscillospirales | Oscillospiraceae |
| b66c5cd65597d8d48b4565e2278a7cd3 | 6.675 | -4.116 | 0.860 | -4.785 | 1.71035E-06 | 0.00059 | Bacteria | Verrucomicrobiota | Verrucomicrobiae | Verrucomicrobiales | Akkermansiaceae |
| 28aff499745e46b984e613d2db705471 | 8.523 | -4.339 | 0.914 | -4.749 | 2.04163E-06 | 0.00066 | Bacteria | Firmicutes | Clostridia | Oscillospirales | Ruminococcaceae |
| b62ac14c1201bce2cc2d7a52bea5e7f7 | 15.102 | 4.930 | 1.043 | 4.728 | 2.26366E-06 | 0.00069 | Bacteria | Campylobacterota | Campylobacteria | Campylobacterales | Helicobacteraceae |
| 32639f00cc49473c7ac97d12a1e64b6e | 19.993 | 5.005 | 1.080 | 4.636 | 3.54831E-06 | 0.00101 | Bacteria | Firmicutes | Clostridia | Oscillospirales | [Eubacterium] coprostanoligenes group |
| ba1410dd9fd0421b26387f18e3a0079d | 11.124 | 4.338 | 0.939 | 4.621 | 3.81337E-06 | 0.00103 | Bacteria | Bacteroidota | Bacteroidia | Bacteroidales | Prevotellaceae |
| 9f1a9fee99e554c172ac91af5f41e970 | 9.285 | 4.432 | 0.971 | 4.566 | 4.97974E-06 | 0.00117 | Bacteria | Verrucomicrobiota | Verrucomicrobiae | Verrucomicrobiales | Akkermansiaceae |
| f1e54f8ff749d3c99c149d3a33d74fc5 | 4.795 | -3.572 | 0.783 | -4.565 | 5.00418E-06 | 0.00117 | Bacteria | Bacteroidota | Bacteroidia | Bacteroidales | Muribaculaceae |
| aebce552a1a4546fee4f0612a89fba94 | 9.369 | -4.451 | 0.973 | -4.573 | 4.81273E-06 | 0.00117 | Bacteria | Bacteroidota | Bacteroidia | Bacteroidales | F082 |
| 266b03a96e692eb75468ec375c827d8d | 12.064 | -4.424 | 0.971 | -4.556 | 5.21408E-06 | 0.00117 | Bacteria | Spirochaetota | Spirochaetia | Spirochaetales | Spirochaetaceae |
| 1f28cce4abd34e22d656ad9c6f1db893 | 4.670 | 3.526 | 0.776 | 4.545 | 5.48747E-06 | 0.00118 | Bacteria | Firmicutes | Clostridia | Oscillospirales | Ruminococcaceae |
| acf08cb2c2051e167ba0c927f0e89888 | 4.513 | -3.470 | 0.770 | -4.503 | 6.69147E-06 | 0.00131 | Bacteria | Bacteroidota | Bacteroidia | Bacteroidales | Prevotellaceae |
| de06d877fe8149feec58b98b682cb0d9 | 5.109 | -3.678 | 0.819 | -4.491 | 7.09528E-06 | 0.00131 | Bacteria | Firmicutes | Clostridia | Clostridia UCG-014 |  |
| c6dfed6474a6b34b6bdb4c498252d057 | 11.249 | 4.137 | 0.922 | 4.489 | 7.14003E-06 | 0.00131 | Bacteria | Firmicutes | Clostridia | Oscillospirales | Oscillospiraceae |
| 7b48569fdfae6abfb874be588b20cb8a | 6.988 | -4.014 | 0.892 | -4.501 | 6.74908E-06 | 0.00131 | Bacteria | Synergistota | Synergistia | Synergistales | Synergistaceae |
| d46abfa0f605c126fc7dbbd5d0a405bf | 15.541 | 4.515 | 1.016 | 4.442 | 8.91159E-06 | 0.00158 | Bacteria | Firmicutes | Bacilli | Erysipelotrichales | Erysipelotrichaceae |
| 69c6251e78f702924ddfcc618f5fcb47 | 11.124 | 4.245 | 0.961 | 4.416 | 1.00588E-05 | 0.00167 | Bacteria | Firmicutes | Clostridia | Oscillospirales | Oscillospiraceae |
| 88f9323e0c3eb3c2500b60a019f2b175 | 5.453 | 3.737 | 0.845 | 4.423 | 9.75357E-06 | 0.00167 | Bacteria | Firmicutes | Clostridia | Clostridia vadinBB60 group |  |
| bb691ff57450e8fc41e733092563dcb8 | 14.194 | 4.537 | 1.033 | 4.394 | 1.11327E-05 | 0.00179 | Bacteria | Bacteroidota | Bacteroidia | Bacteroidales | F082 |
| 5623b47e4e837a8d28fc74bcd562806d | 9.714 | 4.092 | 0.942 | 4.342 | 1.40883E-05 | 0.00220 | Bacteria | Firmicutes | Clostridia | Oscillospirales | Oscillospiraceae |
| 8bda78ffd089c9bd646c8ada1176ab57 | 8.022 | 3.465 | 0.799 | 4.336 | 1.45391E-05 | 0.00220 | Bacteria | Bacteroidota | Bacteroidia | Bacteroidales | Muribaculaceae |
| f482e28108d7c96aae21f5a9535c08e7 | 4.701 | 3.537 | 0.818 | 4.325 | 1.52226E-05 | 0.00224 | Bacteria | Firmicutes | Clostridia | Oscillospirales | Ruminococcaceae |
| c2f141d351d75c66db17400ef0be237d | 5.798 | -3.887 | 0.903 | -4.305 | 1.66928E-05 | 0.00239 | Bacteria | Verrucomicrobiota | Verrucomicrobiae | Verrucomicrobiales | Akkermansiaceae |
| 2bb07523a1d07dcbe1023c32c478aa9a | 10.403 | 3.791 | 0.883 | 4.291 | 1.77556E-05 | 0.00247 | Bacteria | Bacteroidota | Bacteroidia | Bacteroidales | Muribaculaceae |
| 017184872f85f33d5fb749b9a5aee5e5 | 6.174 | 3.625 | 0.850 | 4.266 | 1.99305E-05 | 0.00270 | Bacteria | Firmicutes | Clostridia | Clostridia vadinBB60 group |  |
| ca0afd37a403c122432fd692af338ca2 | 5.077 | 3.604 | 0.848 | 4.247 | 2.16247E-05 | 0.00285 | Bacteria | Spirochaetota | Spirochaetia | Spirochaetales | Spirochaetaceae |
| a8fb6e470552466d3f5112cc711fc635 | 5.171 | 3.537 | 0.838 | 4.224 | 2.40369E-05 | 0.00309 | Bacteria | Verrucomicrobiota | Kiritimatiellae | WCHB1-41 |  |
| 9c625e403e86006e764f181337d048bf | 6.111 | 3.707 | 0.881 | 4.207 | 2.58667E-05 | 0.00325 | Bacteria | Firmicutes | Clostridia | Oscillospirales | Oscillospiraceae |
| 12bac19286f4c2111c9337c8ce3681eb | 4.043 | 3.279 | 0.786 | 4.169 | 3.05479E-05 | 0.00364 | Bacteria | Firmicutes | Clostridia | Oscillospirales | Oscillospiraceae |
| f085d1b218a378c416ad39769a44bbc8 | 5.735 | 3.187 | 0.765 | 4.165 | 3.1087E-05 | 0.00364 | Bacteria | Firmicutes | Clostridia | Eubacteriales | Eubacteriaceae |
| 3e064ac92f41ffcedd02096e322ef0c7 | 4.200 | -3.225 | 0.773 | -4.173 | 3.00025E-05 | 0.00364 | Bacteria | Bacteroidota | Bacteroidia |  |  |
| 2468257d576b300de00e58b2c8fbca61 | 4.388 | 3.265 | 0.787 | 4.151 | 3.30729E-05 | 0.00378 | Bacteria | Proteobacteria | Alphaproteobacteria | Rickettsiales |  |
| 0900cc25c07efdcd88bae4d8c1c9de27 | 6.550 | -3.824 | 0.925 | -4.136 | 3.53803E-05 | 0.00396 | Bacteria | Bacteroidota | Bacteroidia | Bacteroidales | Prevotellaceae |
| 5b95c08dbcb04b1580814df3d2ab21f4 | 9.964 | -3.998 | 0.970 | -4.124 | 3.72834E-05 | 0.00400 | Bacteria | Bacteroidota | Bacteroidia | Bacteroidales | Prevotellaceae |
| 606f24b0a40c81cc8dc94e4567a26c74 | 5.328 | 3.549 | 0.861 | 4.121 | 3.76797E-05 | 0.00400 | Bacteria | Bacteroidota | Bacteroidia | Bacteroidales | Prevotellaceae |
| 83620e9602f29371c62e056000f50686 | 6.205 | 3.614 | 0.879 | 4.114 | 3.88888E-05 | 0.00400 | Bacteria | Firmicutes | Clostridia | Lachnospirales | Lachnospiraceae |
| 2f0f8bb5a02cf145a3fdb7267147d046 | 4.513 | -3.470 | 0.842 | -4.118 | 3.8145E-05 | 0.00400 | Bacteria | Spirochaetota | Spirochaetia | Spirochaetales | Spirochaetaceae |
| e5b567775de40ee2fda74d67d3b5e399 | 7.082 | 3.332 | 0.812 | 4.101 | 4.10514E-05 | 0.00414 | Bacteria | Firmicutes | Clostridia | Oscillospirales | Oscillospiraceae |
| 6af9f488298bb83d53a46b8b0def46ea | 7.865 | 3.121 | 0.765 | 4.081 | 4.48309E-05 | 0.00428 | Bacteria | Campylobacterota | Campylobacteria | Campylobacterales | Campylobacteraceae |
| 137fea548a5dcd6a593f371648360b8b | 3.824 | 3.181 | 0.778 | 4.088 | 4.34551E-05 | 0.00428 | Bacteria | Firmicutes | Clostridia | Oscillospirales | Oscillospiraceae |
| 8fcf8d84a619e76178839c6694515e22 | 11.343 | -3.642 | 0.893 | -4.080 | 4.49599E-05 | 0.00428 | Bacteria | Bacteroidota | Bacteroidia | Bacteroidales | Prevotellaceae |
| c3ae7650e2347b701242dfd01dda2cdb | 4.137 | 3.319 | 0.822 | 4.038 | 5.38268E-05 | 0.00504 | Bacteria | Verrucomicrobiota | Verrucomicrobiae | Verrucomicrobiales | Akkermansiaceae |
| f4a8f9d4e7847bf76aec969bfd077914 | 5.328 | -3.493 | 0.866 | -4.031 | 5.55027E-05 | 0.00510 | Bacteria | Bacteroidota | Bacteroidia |  |  |
| 64ce0486c2676ab1c48ebea626ebbb19 | 3.855 | -3.197 | 0.795 | -4.020 | 5.8248E-05 | 0.00517 | Bacteria | Desulfobacterota | Desulfovibrionia | Desulfovibrionales | Desulfovibrionaceae |
| ab15af2f649be12809aeaa910fb4f077 | 3.794 | 3.168 | 0.788 | 4.020 | 5.82646E-05 | 0.00517 | Bacteria | Verrucomicrobiota | Verrucomicrobiae | Verrucomicrobiales | Akkermansiaceae |
| 4f7ba303e98cdc9c5e33b6790ed5b36b | 4.043 | -3.280 | 0.817 | -4.014 | 5.96081E-05 | 0.00520 | Bacteria | Firmicutes | Clostridia | Oscillospirales | Butyricicoccaceae |
| 8489cc6242e1c04ef37286d13fc0c636 | 4.858 | -3.359 | 0.840 | -3.999 | 6.3498E-05 | 0.00527 | Bacteria | Bacteroidota | Bacteroidia | Bacteroidales | Prevotellaceae |
| 6eab96c069f368a0ca4ba56e7155fed8 | 7.396 | 3.593 | 0.898 | 4.002 | 6.29093E-05 | 0.00527 | Bacteria | Firmicutes | Clostridia | Oscillospirales | Oscillospiraceae |
| 73c5728ff940c770ae15570c6aa24499 | 3.448 | 2.997 | 0.748 | 4.006 | 6.16642E-05 | 0.00527 | Bacteria | Proteobacteria | Gammaproteobacteria | Coxiellales | Coxiellaceae |
| e0c75c48da02ad2f4486b8d9230dd5cf | 3.605 | 3.077 | 0.771 | 3.989 | 6.63347E-05 | 0.00529 | Bacteria | Verrucomicrobiota | Verrucomicrobiae | Verrucomicrobiales | Akkermansiaceae |
| bf4a1f69be2a8455c3b80cf259afa5a2 | 14.914 | -3.985 | 1.000 | -3.987 | 6.68453E-05 | 0.00529 | Bacteria | Verrucomicrobiota | Verrucomicrobiae | Verrucomicrobiales | Akkermansiaceae |
| 64304cb0171f363fd1822cfab4fc153d | 3.981 | -3.253 | 0.815 | -3.992 | 6.54237E-05 | 0.00529 | Bacteria | Bacteroidota | Bacteroidia | Bacteroidales | p-251-o5 |
| 6ef87a3435743b5bcbab03d485b8be17 | 3.981 | -3.239 | 0.814 | -3.980 | 6.89875E-05 | 0.00538 | Bacteria | Proteobacteria | Gammaproteobacteria | Coxiellales | Coxiellaceae |
| 957e4fb40bd831270810bc04a4d43093 | 33.030 | 3.367 | 0.847 | 3.975 | 7.0383E-05 | 0.00540 | Bacteria | Bacteroidota | Bacteroidia | Bacteroidales | Prevotellaceae |
| 6f8638addfffa55ed8010173022e93c7 | 7.208 | 3.537 | 0.892 | 3.968 | 7.25626E-05 | 0.00546 | Bacteria | Firmicutes | Clostridia | Oscillospirales | Oscillospiraceae |
| 850319bff7f0b67cdc76fb310f58a383 | 4.075 | -3.211 | 0.810 | -3.966 | 7.31727E-05 | 0.00546 | Bacteria | Synergistota | Synergistia | Synergistales | Synergistaceae |
| 2a11cd55fc5c35240489d491ed13379a | 7.051 | -3.767 | 0.953 | -3.955 | 7.65739E-05 | 0.00562 | Bacteria | Spirochaetota | Spirochaetia | Spirochaetales | Spirochaetaceae |
| 31cabd090862c648bbeec8557435d4c0 | 5.860 | 3.238 | 0.819 | 3.952 | 7.75428E-05 | 0.00562 | Bacteria | Firmicutes | Clostridia | Oscillospirales | Oscillospiraceae |
| 12e5d46b59d60ae1932ffdf319fc9b5f | 13.348 | 3.747 | 0.952 | 3.935 | 8.30906E-05 | 0.00594 | Bacteria | Bacteroidota | Bacteroidia | Bacteroidales | Muribaculaceae |
| 732cb0f3e76e7f88d7ad992619473eb8 | 5.829 | -3.594 | 0.915 | -3.930 | 8.50762E-05 | 0.00600 | Bacteria | Firmicutes | Negativicutes | Veillonellales-Selenomonadales | Selenomonadaceae |
| 61ea227a617e6edd71bd97f5b163a41d | 3.761 | -3.154 | 0.806 | -3.911 | 9.18639E-05 | 0.00639 | Bacteria | Firmicutes | Clostridia | Oscillospirales | Oscillospiraceae |
| a0c9c49d4d9cd25f45c76b0e3ded4ccc | 4.513 | 3.077 | 0.788 | 3.903 | 9.48592E-05 | 0.00640 | Bacteria | Spirochaetota | Spirochaetia | Spirochaetales | Spirochaetaceae |
| c154ae336d35b0265eea8634438c208a | 3.385 | -2.965 | 0.760 | -3.901 | 9.57524E-05 | 0.00640 | Bacteria | Firmicutes | Clostridia | Oscillospirales | Oscillospiraceae |
| 9f6e814de5bc96631b086e567db9b45e | 4.388 | -3.267 | 0.837 | -3.902 | 9.52257E-05 | 0.00640 | Bacteria | Synergistota | Synergistia | Synergistales | Synergistaceae |
| 3fd19a18daa0b9890d26256dc5d99e27 | 4.764 | -3.346 | 0.861 | -3.886 | 0.000101875 | 0.00672 | Bacteria | Spirochaetota | Spirochaetia | Spirochaetales | Spirochaetaceae |
| 65e8b2b70cdb15805f9a919160c5406e | 4.921 | -3.211 | 0.828 | -3.877 | 0.000105855 | 0.00676 | Bacteria | Bacteroidota | Bacteroidia | Bacteroidales |  |
| 7bd94034cf8e38741081d92d74ffe8b0 | 4.795 | -3.267 | 0.843 | -3.875 | 0.000106501 | 0.00676 | Bacteria | WPS-2 |  |  |  |
| 43fb98332feb62e4c022393b8872150f | 4.952 | 3.319 | 0.855 | 3.880 | 0.00010436 | 0.00676 | Bacteria | Firmicutes | Clostridia | Oscillospirales | Oscillospiraceae |
| 4c386b2df447db461846104a379fb96c | 3.135 | -2.825 | 0.731 | -3.867 | 0.000110355 | 0.00692 | Bacteria | Synergistota | Synergistia | Synergistales | Synergistaceae |
| ba6b8ae0e6acd72c17c204ef94fbe893 | 5.390 | 3.456 | 0.895 | 3.861 | 0.000113055 | 0.00701 | Bacteria | Firmicutes | Clostridia | Oscillospirales | [Eubacterium] coprostanoligenes group |
| 20b15799d8559e451b3193163148c5d3 | 3.135 | -2.825 | 0.732 | -3.857 | 0.000114646 | 0.00702 | Bacteria | Firmicutes | Clostridia | Lachnospirales | Lachnospiraceae |
| 97425d600a442612de22e3c7902ed449 | 5.766 | -3.446 | 0.897 | -3.839 | 0.000123451 | 0.00714 | Bacteria | Firmicutes | Clostridia | Oscillospirales | Ruminococcaceae |
| 383c0ab19cfc0b051736cd571fa24663 | 10.654 | 3.921 | 1.018 | 3.850 | 0.000117931 | 0.00714 | Bacteria | Spirochaetota | Spirochaetia | Spirochaetales | Spirochaetaceae |
| 07c151c6e2f6f7ea2162e9f757bb219c | 7.838 | 3.156 | 0.821 | 3.843 | 0.000121691 | 0.00714 | Bacteria | Bacteroidota | Bacteroidia | Bacteroidales | Rikenellaceae |
| 46e3b1f7c757f374c05c112ea2167800 | 6.487 | 3.614 | 0.940 | 3.844 | 0.000120896 | 0.00714 | Bacteria | Firmicutes | Clostridia | Oscillospirales | Ruminococcaceae |
| 22bad5ed17e1888b51125cbbefcfabdd | 31.393 | -3.745 | 0.975 | -3.841 | 0.00012271 | 0.00714 | Bacteria | Firmicutes | Clostridia | Lachnospirales | Lachnospiraceae |
| c1c57e283677085e4347bb97be5ead3a | 2.980 | -2.730 | 0.713 | -3.829 | 0.000128424 | 0.00721 | Bacteria | Bacteroidota | Bacteroidia | Bacteroidales | Muribaculaceae |
| 8c931f6280edcad1b5bccc899e8691ba | 9.152 | -3.528 | 0.921 | -3.831 | 0.00012781 | 0.00721 | Bacteria | Bacteroidota | Bacteroidia | Bacteroidales | Prevotellaceae |
| c9a75912d7d211881608771843d844c2 | 7.991 | 3.571 | 0.933 | 3.829 | 0.000128867 | 0.00721 | Bacteria | Bacteroidota | Bacteroidia | Bacteroidales | Prevotellaceae |
| a72bbc6c6c7df23fb96fc5288faf6b76 | 3.542 | 3.045 | 0.797 | 3.820 | 0.000133192 | 0.00729 | Bacteria | Firmicutes | Clostridia | Oscillospirales | UCG-010 |
| 312a444296375ec07aec5b9a3c067638 | 3.542 | 3.045 | 0.797 | 3.820 | 0.000133192 | 0.00729 | Bacteria | Firmicutes | Clostridia | Clostridia vadinBB60 group |  |
| f749bb08534f7dbdeaca5938155f783f | 3.229 | -2.879 | 0.759 | -3.794 | 0.000148354 | 0.00756 | Bacteria | Bacteroidota | Bacteroidia | Bacteroidales | Prevotellaceae |
| dc513fb43d3bcd8e4a42654aa3e17a38 | 4.075 | 3.045 | 0.803 | 3.795 | 0.000147772 | 0.00756 | Bacteria | Firmicutes | Clostridia | Oscillospirales | Oscillospiraceae |
| 46f74eb4ad8763a3d3ff64b34f8572fe | 8.053 | 3.122 | 0.824 | 3.789 | 0.000151302 | 0.00756 | Bacteria | Bacteroidota | Bacteroidia | Bacteroidales | Rikenellaceae |
| 8895e0f339e39dfc8ebfcdb764d70cd7 | 8.147 | 3.113 | 0.821 | 3.794 | 0.000148533 | 0.00756 | Bacteria | Firmicutes | Clostridia | Oscillospirales | Oscillospiraceae |
| f1e712ea9d7bcfa146c0a5e7383105ea | 7.145 | -3.458 | 0.909 | -3.803 | 0.000142851 | 0.00756 | Bacteria | Firmicutes | Clostridia | Oscillospirales | Ruminococcaceae |

Table S10. Wilcoxon test for the analysis of the main metabolic pathways.

| Description | .y. | Group1 | Group2 | n1 | n2 | statistic | p-value | p.adj | p.adj.signif |
| --- | --- | --- | --- | --- | --- | --- | --- | --- | --- |
| Calvin-Benson-Bassham cycle | abundance | Treatment0 | Treatment1 | 11 | 11 | 40 | 0.193 | 0.89625 | ns |
| Calvin-Benson-Bassham cycle | abundance | Treatment1 | Treatment2 | 11 | 11 | 73 | 0.438 | 0.89625 | ns |
| L-isoleucine biosynthesis I (from threonine) | abundance | Treatment0 | Treatment1 | 11 | 11 | 37 | 0.133 | 0.89625 | ns |
| L-valine biosynthesis | abundance | Treatment0 | Treatment1 | 11 | 11 | 37 | 0.133 | 0.89625 | ns |
| glycolysis III (from glucose) | abundance | Treatment0 | Treatment1 | 11 | 11 | 45 | 0.332 | 0.89625 | ns |
| glycolysis III (from glucose) | abundance | Treatment1 | Treatment2 | 11 | 11 | 72 | 0.478 | 0.89625 | ns |
| pentose phosphate pathway (non-oxidative branch) | abundance | Treatment0 | Treatment1 | 11 | 11 | 40 | 0.193 | 0.89625 | ns |
| pentose phosphate pathway (non-oxidative branch) | abundance | Treatment1 | Treatment2 | 11 | 11 | 73 | 0.438 | 0.89625 | ns |
| peptidoglycan biosynthesis I (meso-diaminopimelate containing) | abundance | Treatment0 | Treatment1 | 11 | 11 | 45 | 0.332 | 0.89625 | ns |
| peptidoglycan biosynthesis I (meso-diaminopimelate containing) | abundance | Treatment1 | Treatment2 | 11 | 11 | 73 | 0.438 | 0.89625 | ns |
| superpathway of L-threonine biosynthesis | abundance | Treatment0 | Treatment1 | 11 | 11 | 43 | 0.27 | 0.89625 | ns |
| superpathway of L-threonine biosynthesis | abundance | Treatment1 | Treatment2 | 11 | 11 | 72 | 0.478 | 0.89625 | ns |
| superpathway of branched amino acid biosynthesis | abundance | Treatment0 | Treatment1 | 11 | 11 | 42 | 0.243 | 0.89625 | ns |
| superpathway of phospholipid biosynthesis I (bacteria) | abundance | Treatment0 | Treatment1 | 11 | 11 | 42 | 0.243 | 0.89625 | ns |
| tRNA charging | abundance | Treatment0 | Treatment1 | 11 | 11 | 45 | 0.332 | 0.89625 | ns |
| tRNA charging | abundance | Treatment1 | Treatment2 | 11 | 11 | 72 | 0.478 | 0.89625 | ns |
| L-isoleucine biosynthesis I (from threonine) | abundance | Treatment1 | Treatment2 | 11 | 11 | 69 | 0.606 | 0.909 | ns |
| L-valine biosynthesis | abundance | Treatment1 | Treatment2 | 11 | 11 | 69 | 0.606 | 0.909 | ns |
| superpathway of branched amino acid biosynthesis | abundance | Treatment1 | Treatment2 | 11 | 11 | 69 | 0.606 | 0.909 | ns |
| superpathway of phospholipid biosynthesis I (bacteria) | abundance | Treatment1 | Treatment2 | 11 | 11 | 70 | 0.562 | 0.909 | ns |
| Calvin-Benson-Bassham cycle | abundance | Treatment0 | Treatment2 | 11 | 11 | 57 | 0.847 | 1 | ns |
| L-isoleucine biosynthesis I (from threonine) | abundance | Treatment0 | Treatment2 | 11 | 11 | 56 | 0.797 | 1 | ns |
| L-valine biosynthesis | abundance | Treatment0 | Treatment2 | 11 | 11 | 56 | 0.797 | 1 | ns |
| glycolysis III (from glucose) | abundance | Treatment0 | Treatment2 | 11 | 11 | 60 | 1 | 1 | ns |
| pentose phosphate pathway (non-oxidative branch) | abundance | Treatment0 | Treatment2 | 11 | 11 | 62 | 0.949 | 1 | ns |
| peptidoglycan biosynthesis I (meso-diaminopimelate containing) | abundance | Treatment0 | Treatment2 | 11 | 11 | 60 | 1 | 1 | ns |
|  |  |  |  |  |  |  |  |  |  |
| superpathway of L-threonine biosynthesis | abundance | Treatment0 | Treatment2 | 11 | 11 | 60 | 1 | 1 | ns |
| superpathway of branched amino acid biosynthesis | abundance | Treatment0 | Treatment2 | 11 | 11 | 55 | 0.748 | 1 | ns |
| superpathway of phospholipid biosynthesis I (bacteria) | abundance | Treatment0 | Treatment2 | 11 | 11 | 58 | 0.898 | 1 | ns |
| tRNA charging | abundance | Treatment0 | Treatment2 | 11 | 11 | 59 | 0.949 | 1 | ns |


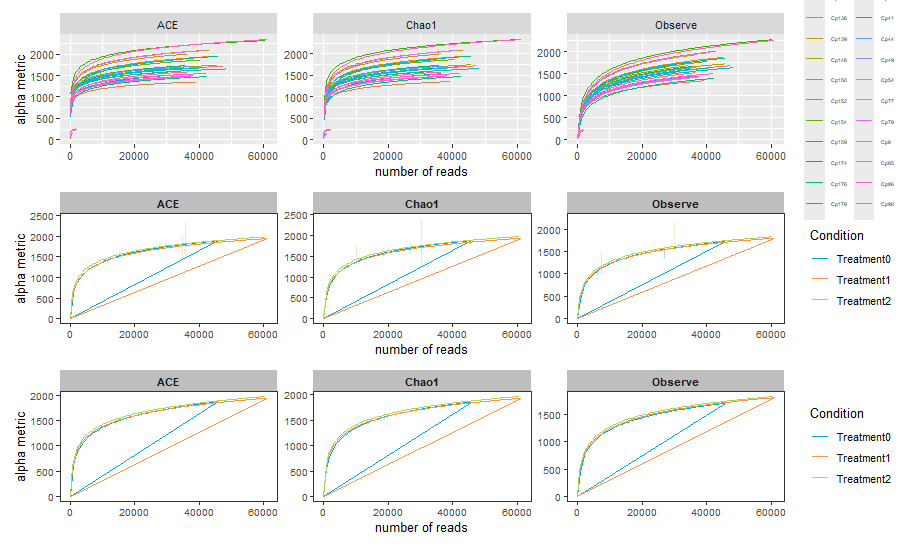
Fig. S1. Rarefraction curve analysis for ACE, Chao1 and Observe indices, corresponding to the MicrobiotaProcess library.

Fig. S2. Box Plot of statistical significance for the Bray-Curtis index, using the PERMANOVA test.


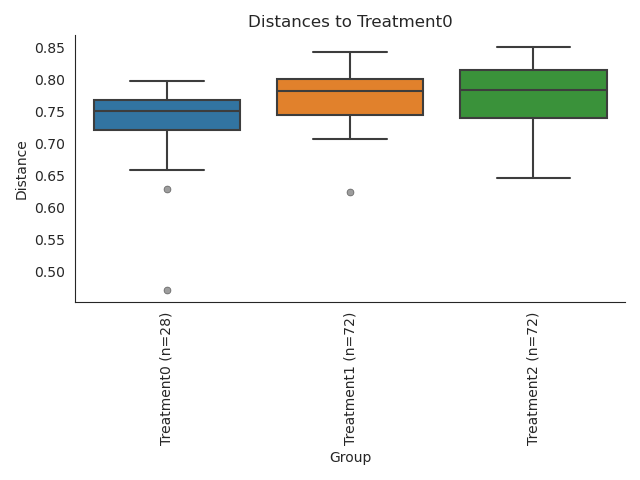

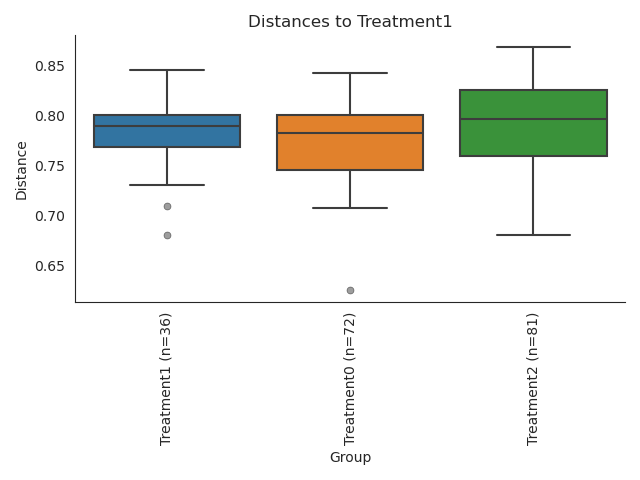

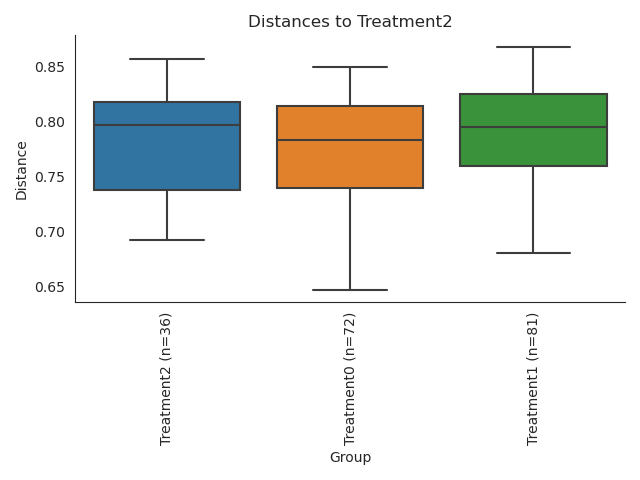


Fig. S3**.** Box Plot of statistical significance for the Jaccard distance index, using the PERMANOVA test.


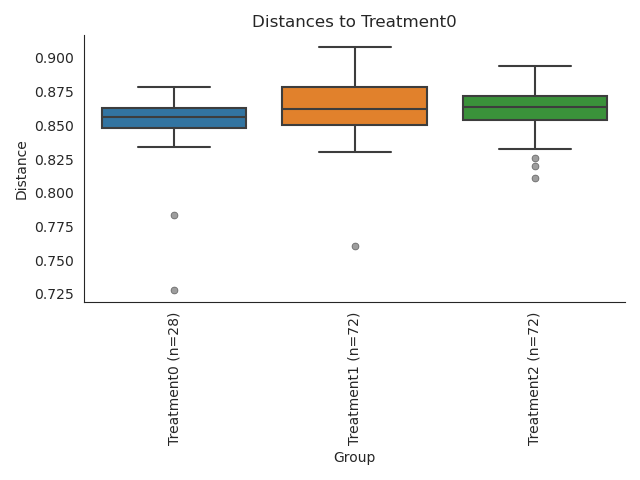

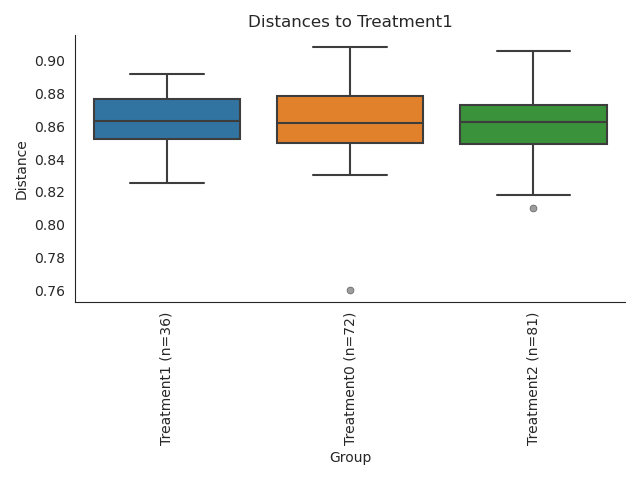

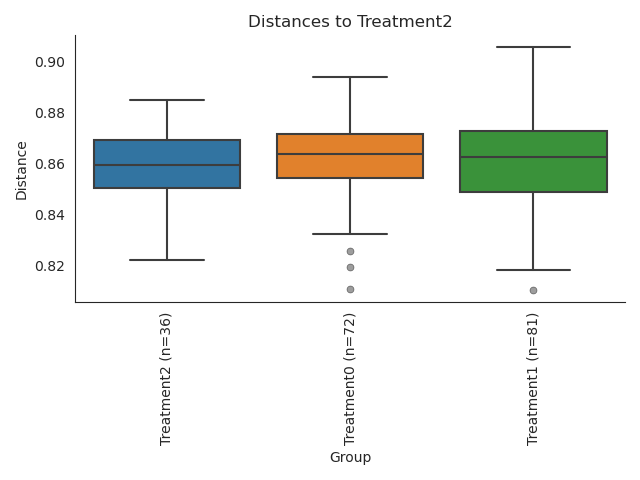


Fig. S4. Box Plot of statistical significance for the Weighted Unifrac distance, using the PERMANOVA test.


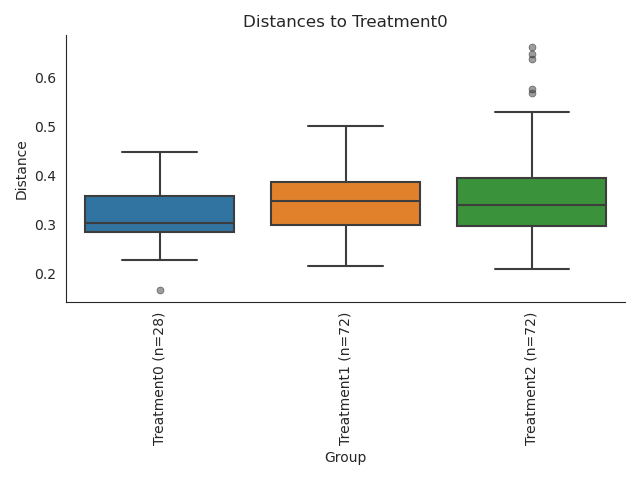

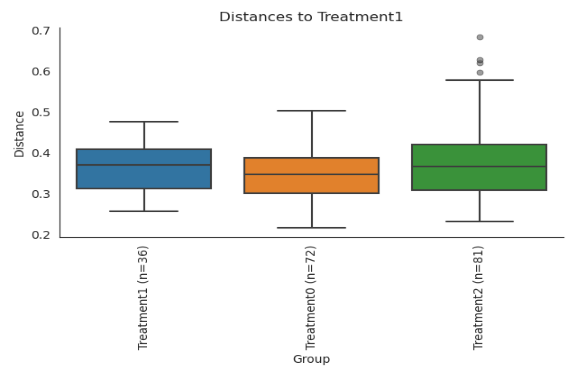

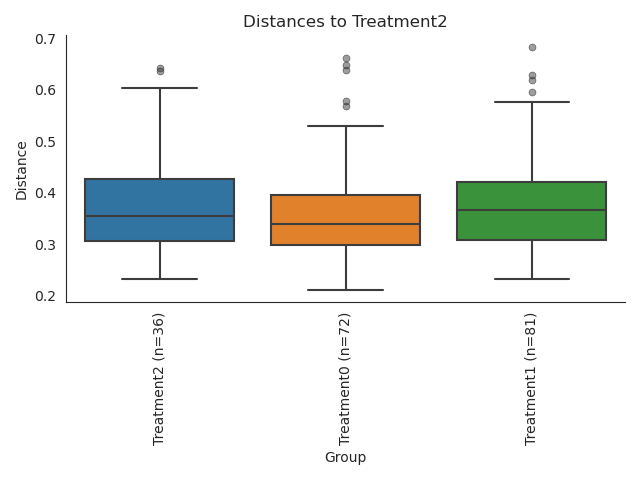

Fig. S5. Differential taxonomic composition of guinea pig cecal microbiota between treatments at the Phylum level, calculated with the MicrobiotaProcess library.


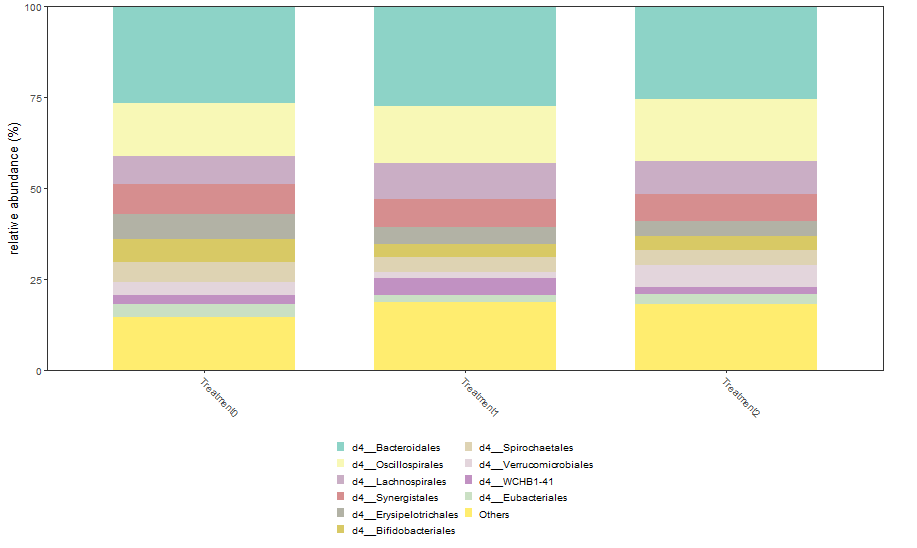
Fig. S6. Differential taxonomic composition of guinea pig cecal microbiota between treatments at the Order level, calculated with the MicrobiotaProcess library.


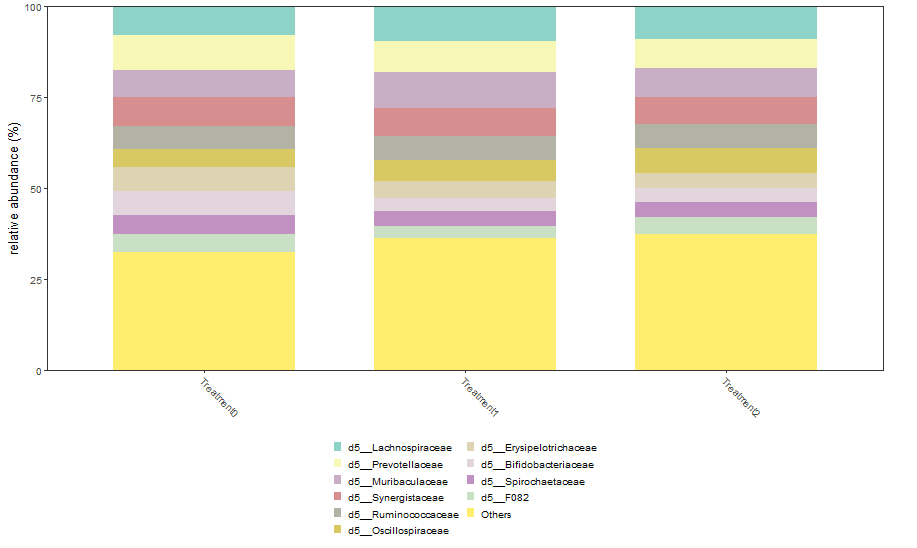
Fig. S7. Differential taxonomic composition of guinea pig cecal microbiota between treatments at the Family level, calculated with the MicrobiotaProcess library.


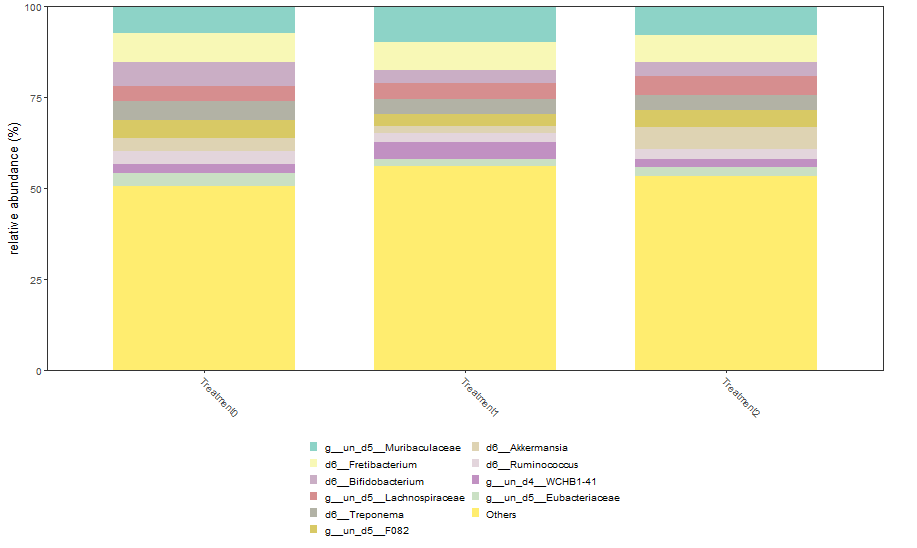
Fig. S8. Differential taxonomic composition of guinea pig cecal microbiota between treatments at the Genus level, calculated with the MicrobiotaProcess library.
